# Scoliosis and Lower Limb Inequality: To Lift or Not to Lift, That Is the Question

**DOI:** 10.7759/cureus.58443

**Published:** 2024-04-17

**Authors:** Saverio Colonna, Fabio Casacci, Corrado Borghi

**Affiliations:** 1 Rehabilitation Medicine, Spine Center, Bologna, ITA; 2 Research and Development, Osteopathic Spine Center Education, Bologna, ITA

**Keywords:** coronal malalignment, pelvic obliquity, lower limb discrepancy, heel lift, coronal balance, pelvic asymmetry, lower limb inequality, scoliosis

## Abstract

In subjects with scoliotic alterations of the spine, asymmetrical lengths of the lower limbs are frequently observed, a condition commonly referred to as leg length inequality (LLI) or discrepancy (LLD). This asymmetry can induce pelvic misalignments, manifested by an asymmetric height of the iliac crests, and consequently an alteration of the spine's axis. Although correcting this discrepancy might appear to be a straightforward solution, further investigation may reveal other indications.

The purpose of this article is to aid clinicians confronted with the decision of whether to compensate for an LLI in individuals with scoliosis, encompassing both adolescents and adults. It presents a literature review on the incidence of LLIs in the general population, distinguishing between structural LLI (sLLI) and functional LLI (fLLI) types of LLIs, and quantifying their magnitude with clinical and instrumental evaluation. Additionally, it links these two types of LLIs to the type of scoliosis (structural or functional).

From a clinical perspective, it also examines the compensatory mechanisms employed by the pelvis in the presence of structural or functional LLIs in order to draw useful indications for therapeutic decisions.

Moreover, it proposes an additional evaluation parameter in the coronal plane, namely the central sacral vertical line (CSVL), to aid in the decision-making process regarding LLI compensation. Although this parameter has been documented in the literature, it has been little associated with LLIs. The findings indicate that scoliotic discrepancies should be compensated (conservatively or surgically) only when the imbalance of the femoral heads is on the same side as the imbalance of the sacrum and the iliac crests; this corrective action should result in a reduction of the overhang in the coronal plane.

## Introduction and background

Definitions

Definition of Lower Limb Inequality

The term leg length inequality (LLI) or discrepancy (LLD) refers to a difference in length between the two limbs, which is a frequent occurrence in the population. In fact, it often happens that heterometry of the lower limbs is highlighted during a pediatric or orthopedic check-up [1].

Etiology of Structural and Functional LLI

LLIs are divided into two etiological groups: structural LLIs (sLLIs) are associated with a shortened bony structure constituting the lower limb, whereas functional LLIs (fLLIs) are dependent on the altered mechanics of the lower extremities [1].

Categories of People with LLI

People with an LLI are classified into two categories: those who have had an LLI since childhood and those who developed an LLI later in life. Concerning functional outcomes like gait, individuals who acquire an LLI in later life experience greater debilitation from an LLI of equivalent magnitude compared to those who have had this condition since childhood [2].

Reasons for the Occurrence of sLLI

The reasons for the occurrence of a structural difference are unknown in 95% of cases [3-5]. Of the recognized factors, two primary causes merit differentiation: processes directly altering the bone length and those inducing asymmetrical growth [3,5]. The first group includes fractures of the diaphysis of the lower limb with an incomplete union or growth disorders, congenital or acquired bone deformations, and deformations of the hip joint, including iatrogenic issues, such as those that occur after hip joint arthroplasty [6,7]. The second group includes inhibition or stimulation of limb growth on one side. Inhibition of limb growth may result from an epiphyseal injury or may be related to paralysis, inflammation, ischemia, tumors, necrosis, or congenital deformation of the extremities [8].

Reasons for the Occurrence of fLLI

An fLLI may be caused by an alteration of lower limb mechanics, such as joint contracture, static or dynamic mechanical axis malalignment, muscle weakness, or shortening. fLLI can develop due to an abnormal motion of the hip, knee, ankle, or foot in any of the three planes of motion [9].

## Review

Prevalence of LLIs

A moderate and asymptomatic difference in the length of the lower limbs is common. A dated study [9] reports that approximately 15% of the adult population has an LLI greater than 1 cm, whereas Friberg [10] believes that approximately half the population presents an LLI of 0.5 cm, which is associated with an increased prevalence of low back pain. Epidemiological researchers have found that an LLI within 2 cm affects between 40% and 70% of the adult population [11,12]. In our research, we did not find studies on the incidence of LLI in the juvenile or adolescent population.

**Figure 1:**
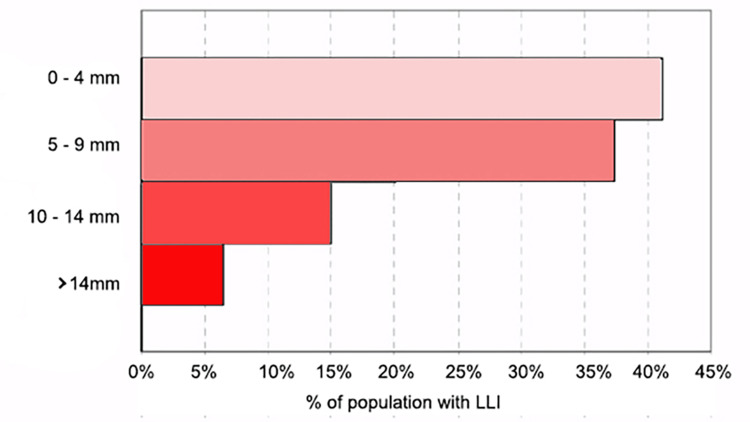
Distribution of the prevalence of LLIs. The distribution of the prevalence of LLIs in a sample of 2978 healthy subjects reported in Knutson's review [13], where however the age of the subjects evaluated was not reported. LLI: leg length inequality Adapted from Knutson [13]

In a 2005 systematic review [13], Knutson and other researchers used leg length data obtained with accurate and reliable radiographic methods from eight studies that together comprised a sample of 2978 healthy subjects, where the age of the subjects evaluated was not reported; their results indicate that only 10% of the adult population examined had equal length of the lower limbs (Figure 1). The mean extent of anatomical inequality was 5.2 mm (standard deviation (SD) = 4.1), and an LLI equal to or less than 1 cm was found in almost 90% of the population. The authors conclude that anatomical LLI is nearly universal but the average magnitude is minimal and probably clinically insignificant. In the same study, the authors report that there is no gender difference in the incidence of LLI, and the relationship with height is doubtful.

LLI evaluation methods

Although imaging techniques are believed to be the most accurate method for determining LLIs, they are expensive and time-consuming; furthermore, X-rays and CT scans expose the patient to harmful radiation. Therefore, in this study, alternative clinical methods were used.

Clinical evaluation

**Figure 2:**
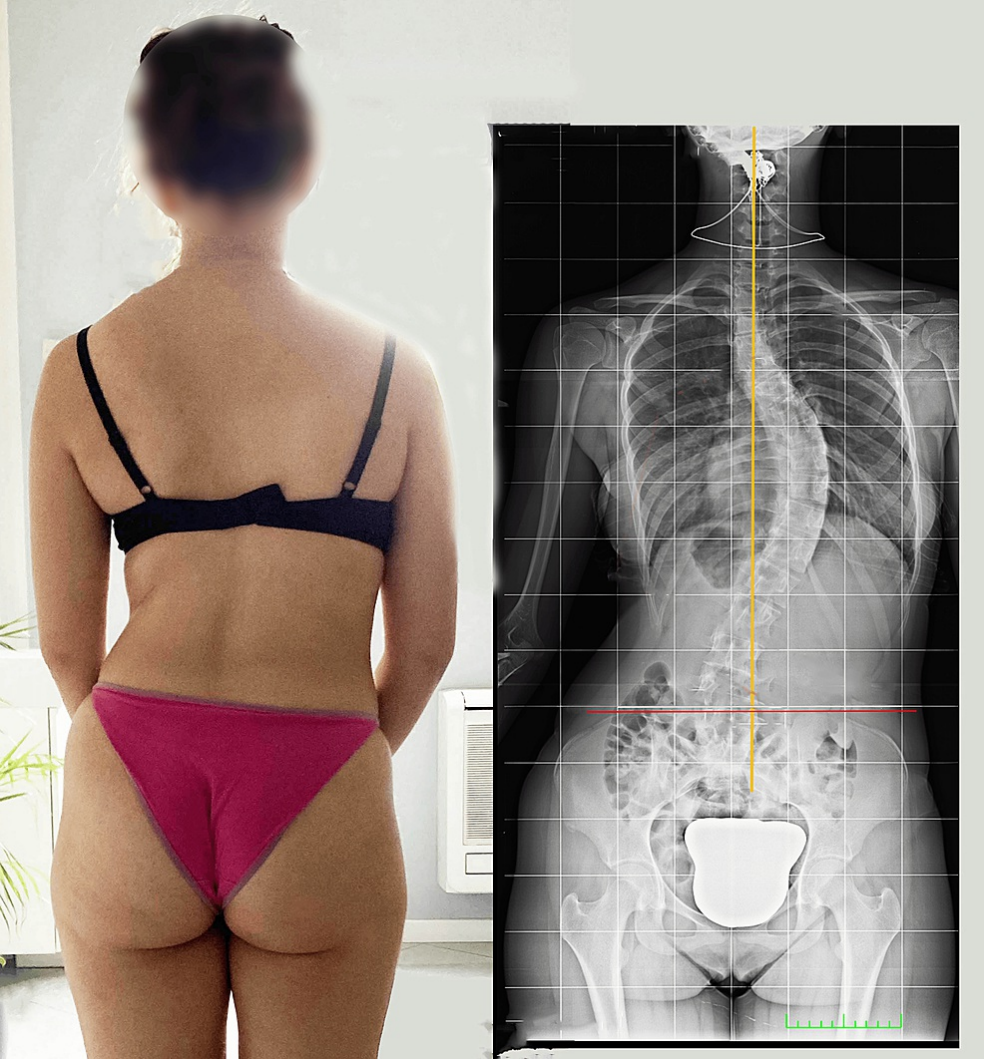
A scoliotic female subject and a relative radiographic image of her pelvis and spine. On the left, this scoliotic patient presents with visible asymmetry of the ridges; on the right, the same subject's X-ray illustrates that the iliac crests are level. Image Credit: Author Saverio Colonna

For evaluations of LLI, simple observation can often lead to errors; this occurred in the case reported in Figure 2. Several authors have noted that it is a mistake to assume that the side and amount of an LLI can be reliably inferred from the malalignment of the iliac crests [14,15].

**Figure 3:**
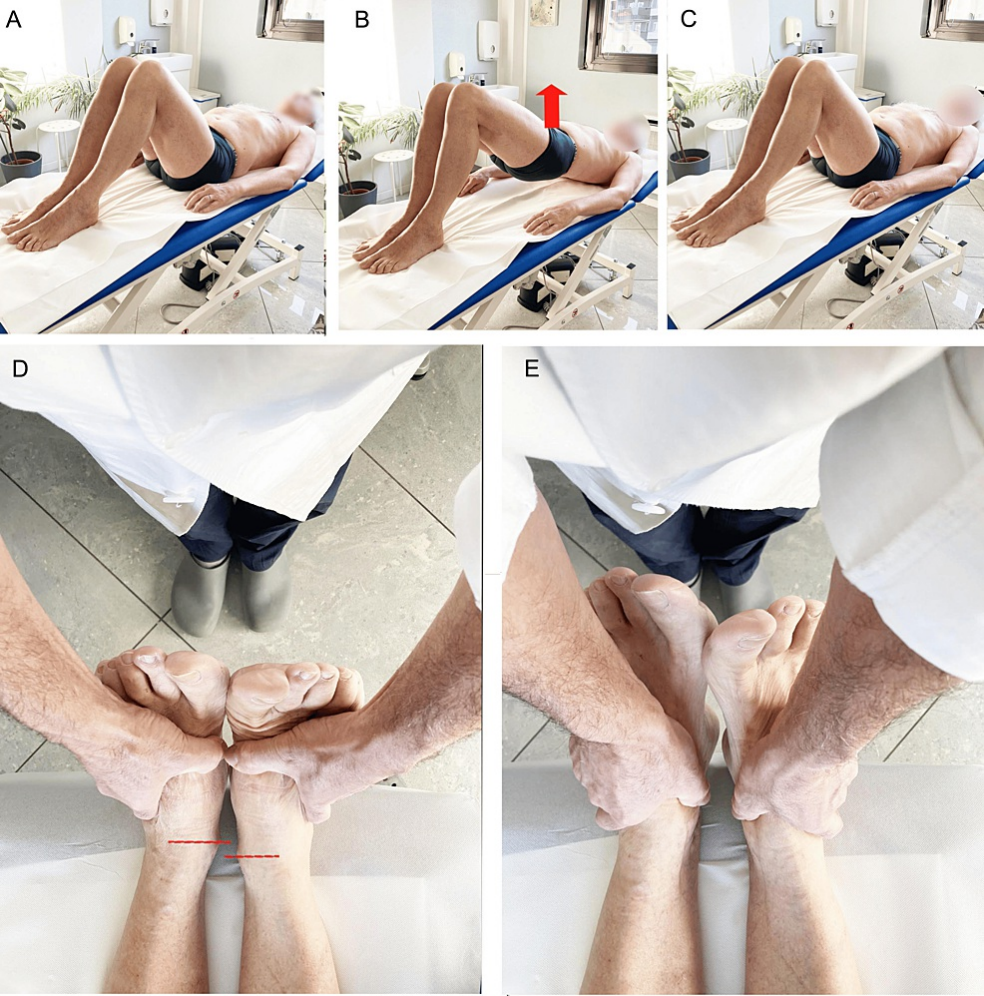
Weber-Barstow maneuver. The top row of photographs (A, B, C) demonstrates an example of the implementation [16] of recommended movement to rebalance the pelvis; the bottom row of photographs (D, E) illustrates methods to find the level of the malleoli [17]. Image Credit: Author Saverio Colonna

The most commonly used qualitative method to evaluate LLI is the Weber-Barstow maneuver (Figure 3), which is conducted with the subject in a supine position. The examiner holds the subject's ankles, placing a thumb under each malleolus, visually comparing the internal edges of both malleoli. Following this, the patient performs a maneuver to relax the pelvic muscles and lower limbs: by flexing the hip and knee, the patient lifts the pelvis, breathes through the nose, remains in this position for 10 seconds, and then slowly lowers the pelvis. At this point, the examiner lengthens the lower limbs to again visualize the levels of the internal border of both malleoli [16].

Another method, proposed by Magee [17], involves the patient in a supine position with extremities in an extended position; the examiner, holding the patient's ankles, places the thumbs under the malleoli and visually compares the internal edge of both medial malleoli (Figure 3). If the examiner notices a difference in leg lengths that persists after asking the patient to sit, then an sLLI is indicated; if, however, the sitting position reduces the length difference until it disappears, then this indicates an fLLI. The rationale behind this test is that an fLLI due to pelvic torsion affects the length of the limb to a lesser extent in the sitting position than in the supine position.

For a quantitative evaluation of LLI, two methods of clinical measurement have been established.

**Figure 4:**
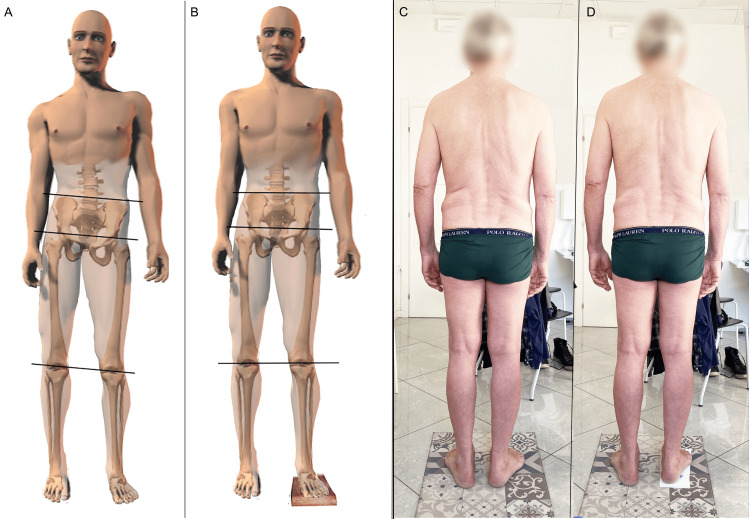
Indirect method to verify limb length. These images demonstrate a search for symmetry by using the iliac crests and artificially lengthening the shorter limb by using a lift under the foot. The illustrations on the left offer a diagram of pelvis realignment in a patient with an sLLI by placing a block under the foot of the shorter limb; the photographs on the right feature a subject with an LLI before and after lifting the right foot by 10 mm. LLI: leg length inequality; sLLI: structural leg length inequality Image Credit: Author Saverio Colonna

a) The indirect method is performed with the patient in a standing position; the examiner compares the level of the pelvis before and after placing lifting blocks under the foot of the shorter leg and visually examines the level of the pelvis [18] (Figure 4).

**Figure 5:**
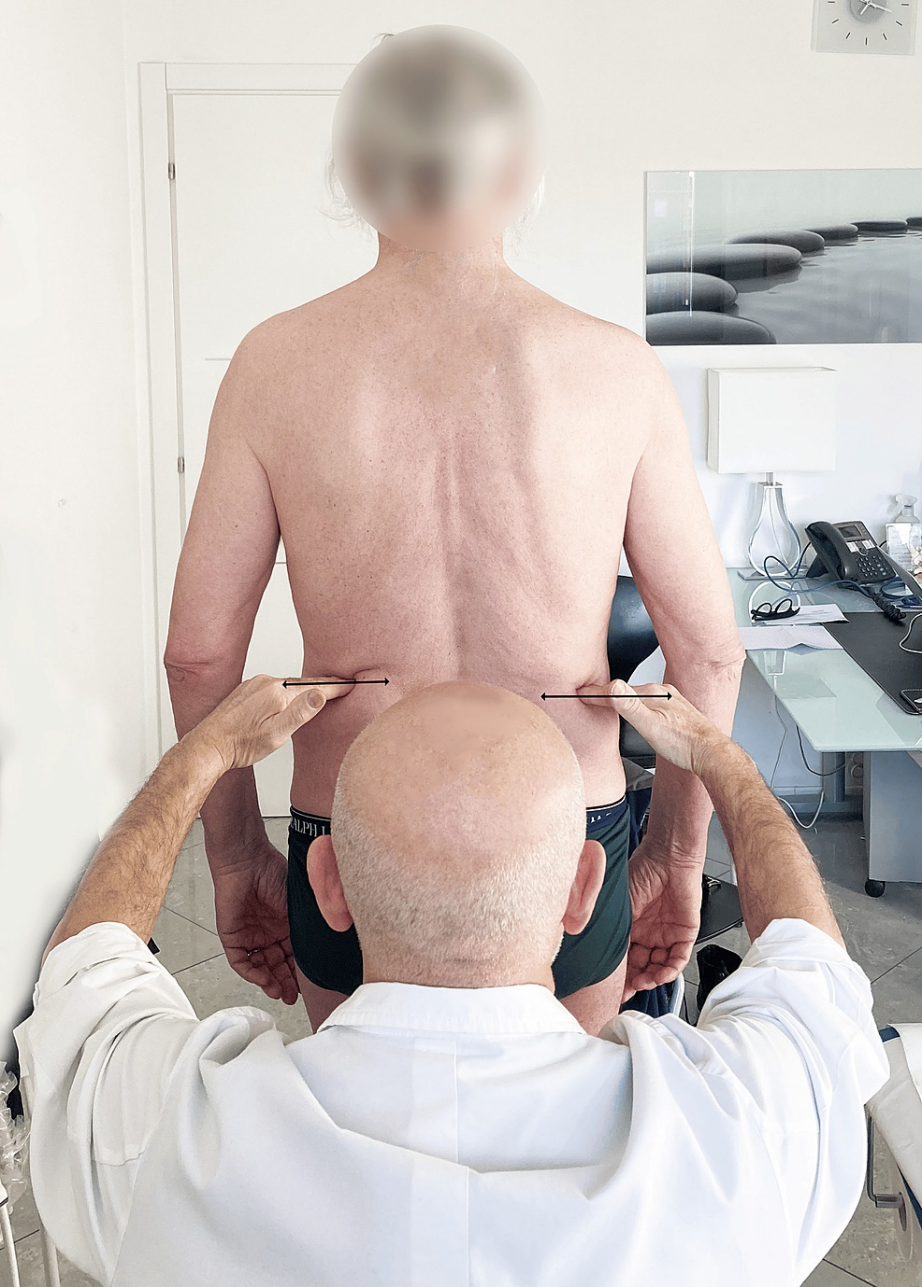
Manual assessment of iliac crests' heights. Demonstrating how to take the heights of the iliac crests to perform an indirect assessment of the length of the lower limb. Image Credit: Author Saverio Colonna

The evaluation can be performed either visually directly on the body of the examinee, or by placing the tips part of the fingers of the hands on the apex of the lateral portion of the iliac crest, to better highlight the height of the crests, and visually the operator evaluates the heights of his hands [18] (Figure 5). The indirect or standing method, unlike the direct one, incorporates the contribution of the foot and ankle to the length of the limb.

**Figure 6:**
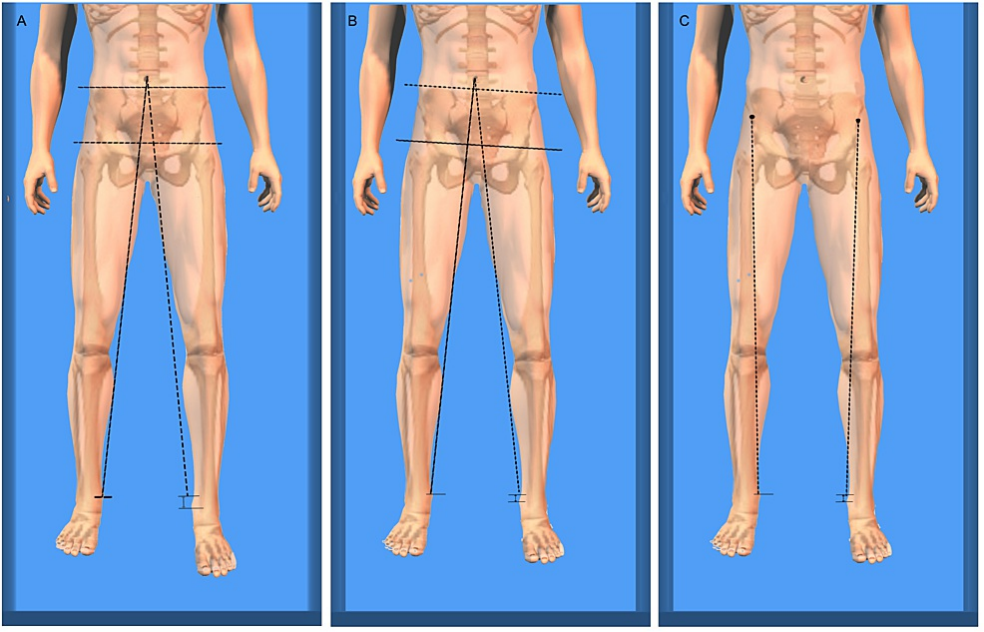
The direct method of measuring, in the supine position, the distance of the fixed bony reference points with a tape measure. (A): sLLI cases feature a real difference in the length of the lower limbs. When the limbs are extended in a neutral position, the level of the malleoli is different. In fLLI (B), as determined by the presence of pelvic obliquity, there appear to be different lengths of the limbs, (C) although, in reality, the measurement from the superior iliac spines to the malleoli demonstrates the equal length of the limbs [18]. sLLI: structural leg length inequality; fLLI: functional leg length inequality Image Credit: Author Saverio Colonna

b) The direct method is performed, with the patient in the supine position, by using a tape measure to note the distance between the fixed bony reference points. In the literature, using a tape measure to note the distance from the umbilicus to the lower edge of the medial malleolus has been proposed (Figure 6) [19], but this measurement remains approximate because of the positional mobility of the umbilicus.

Two other measurements that use a tape measure are commonly employed: the distance between the anterosuperior iliac spine (ASIS) and the median malleolus as well as the distance between the ASIS and the lateral malleolus [20].

Rondon et al. [21] compared the clinical methods (true distance from the ASIS to median malleolus and apparent distance from umbilicus to median malleolus) of measurements of LLI with radiographic measurement in 17 adult patients. Despite high interobserver reliability of the true (intraclass correlation coefficient (ICC), 0.99) and apparent (ICC, 0.88) measurement of clinically assessing LLI, the concordance between the true measurement and radiographic assessment (ICC, 0.80) and apparent method and radiographic assessment (ICC, 0.75) was lower. However, some conditions may limit this manual method's validity in clinical use, such as the difficulty in correctly identifying the bony landmarks or angular axis alterations due to excessive body fat. Additionally, some causes of LLI, such as peroneal hemimelia and post-traumatic bone loss that involve the foot, produce a significant portion of limb shortening below the tibial mortice. Measuring from the pelvis to the bottom of the calcaneus [22] is, therefore, more easily reproducible and can account for the shortening distally to the ankle; additionally, this method reproduces a truer measurement of the entire lower extremity and is more accurate. In many cases, apparent shortening may result from pelvic obliquity or contractures around the hip and knee joints, although the length of the limbs is the same [22].

**Figure 7:**
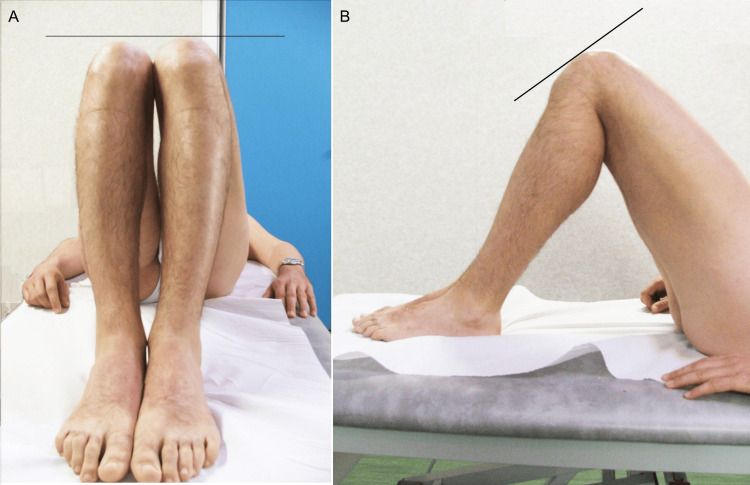
Galeazzi sign in a patient with an LLI. The direct method of evaluating the length of the femur, tibia, and foot [23]. LLI: leg length inequality Image Credit: Author Saverio Colonna

If an LLI is suspected from the pelvic inclination while the patient is standing, then the location of the discrepancy can be verified by performing the Galeazzi sign (Figure 7), also called the Allis or Skyline test, which is performed with the patient in a supine position; the examiner notes the relative heights of the knees when both hips and knees are flexed at 90° and the feet are on a level surface [23,24].

When tested, a higher kneecap is indicative of a longer tibia bone, whereas a more distal tibial tuberosity indicates a longer femur bone. A further evaluation of the length of the tibia and foot can be conducted by comparing the height of the sole of the heel while the patient is prone, knees and ankles are flexed at 90°, and both hips are in a neutral position.

These clinical tests cannot be performed if significant contractures are present that limit hip, knee, or ankle movement. When used correctly in combination, the direct manual assessment of the iliac crests' heights in a standing position (Figure 5) and Galeazzi tests can provide a reasonable clinical estimate of an LLI.

**Figure 8:**
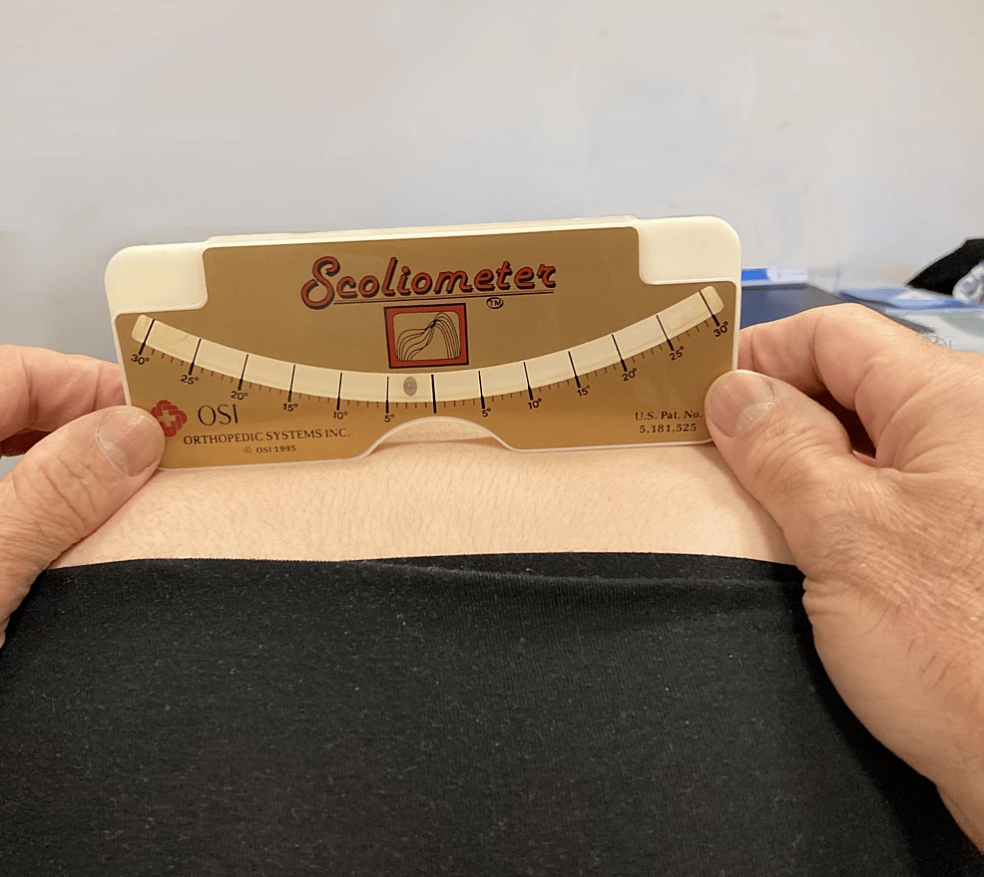
Example of an LLI assessment using a scoliometer positioned on the PSIS. In the patient tested, a leveling of approximately 3° is noted with a minus on the left side. LLI: leg length inequality; PSIS: posterosuperior iliac spine Image Credit: Author Saverio Colonna

The examiner may misdiagnose an sLLI when the patient has an fLLI resulting from an angular limb deformity or a joint contracture of the lower limb, as is the case for the patient portrayed in Figure 2. For example, the patient may have symmetrical bone segment lengths, but one may appear shorter if there is a unilateral flexion contracture of the knee or a valgus contracture of the hip and knee or ankle and foot. The lower limb may appear to be overly long if the Achilles tendon is contracted, thus causing the heel to lift. A level pelvis can be verified indirectly using a scoliometer to evaluate the sacral hump or directly on the posterosuperior iliac spine (PSIS) during the trunk flexion portion of Adam's test (Figure 8) [25].

There is considerable disagreement in the literature regarding the validity and reliability of clinical methods. While people continue to be taught to use tape measures to note from the malleoli to the umbilicus or the ASIS, this method often results in inaccurate estimates of discrepancy [18,26,27].

Friberg et al. [28] evaluated 196 subjects for lower limb asymmetry using direct and indirect methods; furthermore, they compared clinical assessments with radiological measurements in 21 scoliotic patients. The clinical methods proved to be inaccurate and significantly imprecise: the average observer error was +/- 8.6 mm for direct measurement and +/-7.5 mm for indirect measurement. More than half (53%) of the observations were incorrect when the criterion for LLI was 5 mm. Failure to determine the presence or absence of inequality greater than 5 mm in length occurred in 54 measurements (27% of the total). In 12% of the direct and 13% of the indirect measurements, observers mistakenly decided which leg was longer; discrepancies also occurred when the radiological reading provided an inequality in leg length of up to 25 mm.

Cleveland et al. [26] compared, in 10 patients, tape measurements of LLIs with standing and supine radiographs; they reported a poor-to-moderate correlation. Clarke [29] tested the indirect method against radiographs; two examiners measured within 5 mm of the radiographic length in only 16 of the 60 subjects assessed. Mann et al. [30], furthermore, studying the agreement for palpation and observation of iliac crest heights revealed poor reliability in determining the heights of the iliac crests, which is a fundamental requirement of the indirect method. Palpating the iliac crests to determine whether they are level may be less reliable in obese patients [31] or in children with significant trunk asymmetry caused by lumbar scoliosis.

Gross et al. [15] used the indirect method with a device built ad hoc to evaluate pelvic leveling, reporting good intra-examiner reliability (ICC = 0.84) and fair inter-tester reliability (ICC = 0.77) and validity compared to the radiographic gold standard, which is between 0.55 and 0.76.

Consistent with this finding, Beattie et al. [20] found that clinical direct detection validity estimates of LLIs had an ICC of r = 0.683 when using a single measurement of the ASIS and medial malleolus; however, the mean of two measurements (test and retest) taken a few minutes apart demonstrated an improved validity of r = 0.793. Conversely, Gogia and Braatz [32] reported, after using radiographs, an inter-examiner of direct detection reliability of r = 0.98 and a validity (ICC) of r = 0.98.

The results from Hoyl et al. [33] align with previous work; in fact, they report an intra-tester reproducibility, for the evaluation of the distance between the ASIS and the medial malleolus, between r = 0.89 and 0.95 as well as an inter-tester reproducibility of r = 0.96; however, the comparison between the clinical evaluation and the instrumental evaluation with Metrecom device (not with the radiographs) highlights a statistically significant difference.

To redeem this controversy on the validity and repeatability of clinical assessments of limb length, the systematic review work of Farahmand et al. [34] concluded:

“Overall, according to studies in which the validity and reliability of tape measure assessment have been evaluated, this method has been shown to exhibit acceptable reliability and validity among healthy individuals. However, it fails to show acceptable validity for obese individuals or those with orthopedic problems. The examiner should have the skill and experience necessary to find bony landmarks and correctly perform lower extremity measurements. Such a method may provide unrealistic results for those who have pelvic tilt or other problems; therefore, it is necessary to use a method suitable for both healthy and obese patients. Furthermore, the method should be performed in a standing position, should eliminate the need for x-rays, and should not require the special skill and experience of the examiner."

In the literature [18], the indirect and direct methods are compared with radiographs; researchers have reported that the indirect method is more accurate and precise than both direct methods. Of the two direct methods, the ASIS measurement at the lateral malleolus is more accurate than the measurement taken at the medial malleolus. Researchers [18] have therefore recommended using the indirect method in cases where there may be a functional discrepancy. Woerman and Binder-Macleod [18] proposed, for patients who can maintain a neutral standing position, measuring the iliac crests' levels clinically with the radial edge of the index finger (see Figure 5); alternatively, inserting wooden blocks or part of an old telephone book under the foot of the shorter limb until the ridges are symmetrical allows examiners to quantify the discrepancy. This assessment, which seems simple, is difficult to perform because the iliac crests have a curvilinear appearance; therefore, finding the same point bilaterally is complex; furthermore, the layer of fat often present in this area does not facilitate bone contact.

Raczkowski et al. [35] also propose sampling the PSIS for the palpatory evaluation of limb symmetry, believing it to be more reliable. It must be underscored, however, that this bone landmark has poor inter- and intra-examiner reliability in palpatory sampling [36].

LLI and lumbar lateral flexion

**Figure 9:**
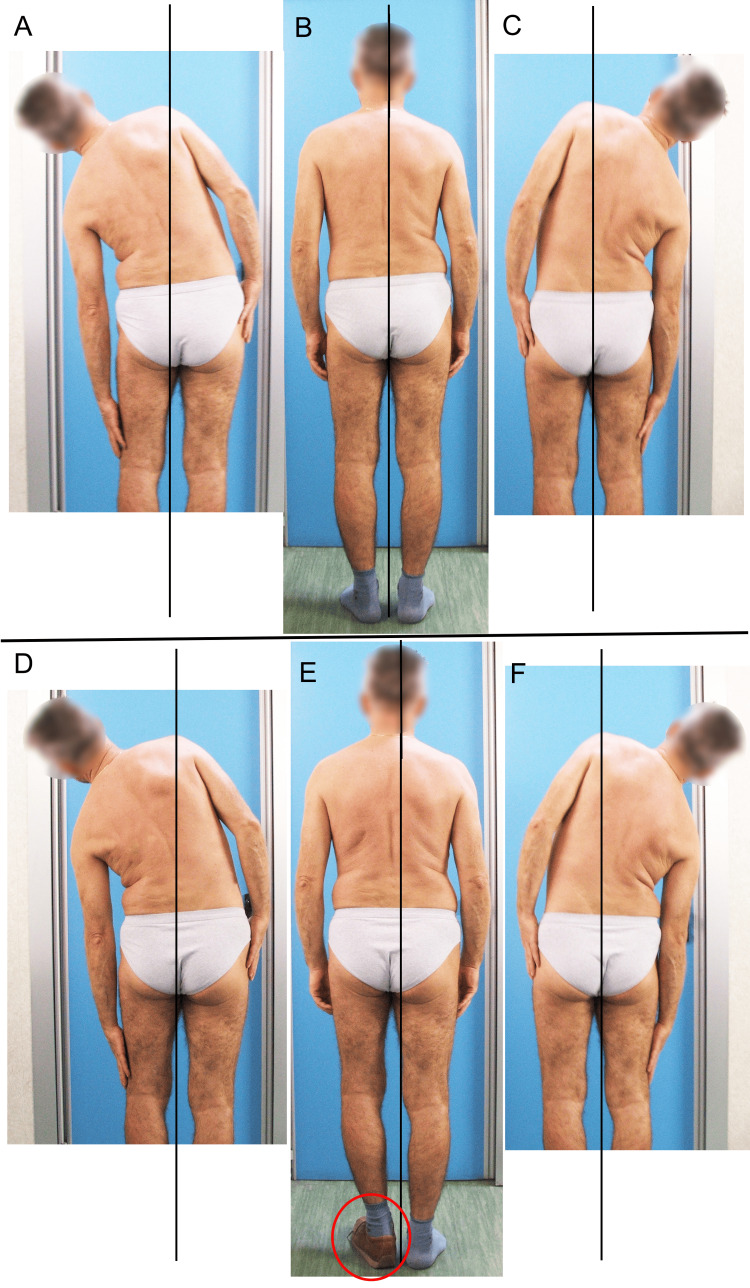
This subject acquired LLI after bone maturity due to a femoral fracture. A patient with the left limb (on X-rays appears to be approximately 23 mm) shorter than the right. In the top row of images, the photograph on the left (A) features the patient demonstrating maximal left flexion; in the center (B), the neutral upright posture; and on the right (C), maximal right flexion. Less lateral flexion on the side of the longer limb is clinically evident. The bottom row of images (D, E, F) presents the same poses by the same subject wearing on the left foot a shoe with a heel of approximately 20 mm. A comparison of the two scenarios indicates that wearing the shoe allows a greater symmetry of the lateral flexion between the two sides. Image Credit: Author Saverio Colonna

To identify whether an LLI is structural or functional and deserving of compensation, evaluating spinal mobility in lateral flexion can also be useful. Researchers studied lumbar lateral flexion [37] in a group of patients examined 10 years after developing an LLI caused by a femoral fracture; the LLI additionally occurred after skeletal maturity. Clinical assessment of lateral bending of the spine before the correction of leg inequality revealed a greater range of movement toward the shorter leg (Figure 9). Paradoxically, because of preexisting sacral tilt, this was not reflected in the radiographic measurement of lateral flexion where mean lateral flexion toward the short leg was lesser.

The subjects, in spite of compensatory lumbar scoliosis resulting from the fracture, had more or less symmetrical lumbar lateral flexion after compensation, compensation such as a shoe insert only on the shortest limb (Figure 9). The authors [37] suggest that the acquired limb length discrepancy produced a small permanent structural abnormality in the lumbar spine. Significant anatomical LLI acquired after skeletal maturity seems not to translate into adaptive structural changes within a 10-year period. Researchers in another study [38], however, examined the effects of a significant LLI (average 3 cm) in now-mature subjects (an average age of 28 years) who acquired the LLI before skeletal maturity. They found a notable lumbar lateral flexion asymmetry, even when the LLI was compensated, with the greatest width on the side of the longer limb where the concavity of the lumbar curve was present. It should be emphasized that in that study, lateral bending was assessed clinically with a flexible tape measure to record the distance of markers drawn on the flank of the tested subjects before and after placing a lift under the shorter limb to balance the pelvis. Nevertheless, this suggests that the body permanently compensates for the structural changes in the spine and pelvis.

**Figure 10:**
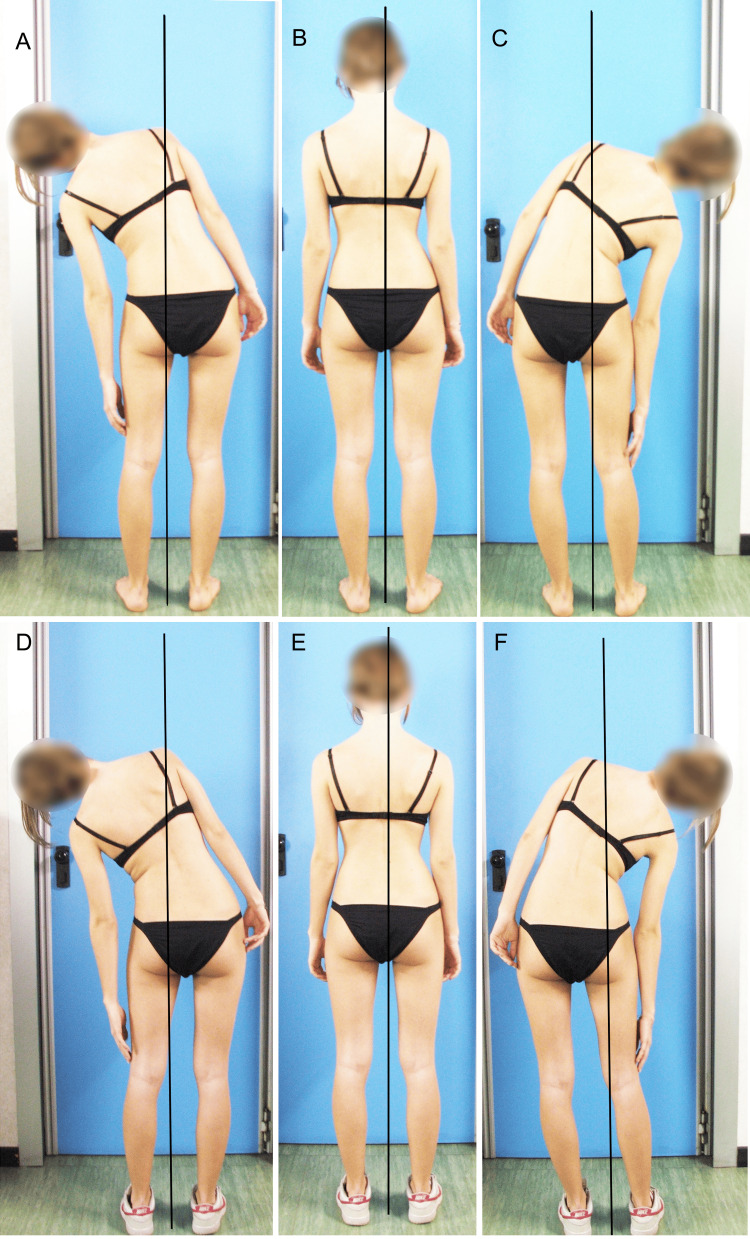
This subject acquired idiopathic LLI before bone maturity. In the top row of images, the photograph on the left (A) features the patient demonstrating maximal left flexion; in the center (B), the neutral upright posture; and on the right (C), maximal right flexion. The bottom row of images (D, E, F) features the same patient in the same poses; however, the patient now wears shoes with a 10 mm high insole in the left shoe. Image Credit: Author Saverio Colonna

Figure 10 illustrates an example of lateral flexion of a subject with a mild LLI resulting from the femur fracture; the fracture occurred 22 years earlier: the left limb is approximately 15 mm shorter than the right and the patient has consequent left convex scoliosis. In the top row of photos, the patient is barefoot; in the bottom row, the patient wears the shoe, approximately 10 mm in height, only on the shortest limb. As can be seen, the symmetry of flexion between the two sides improves with the shoes, although a slight deficit remains on the side of the shorter limb; Papaioannou et al. [38] highlighted this discrepancy in their work.

**Figure 11:**
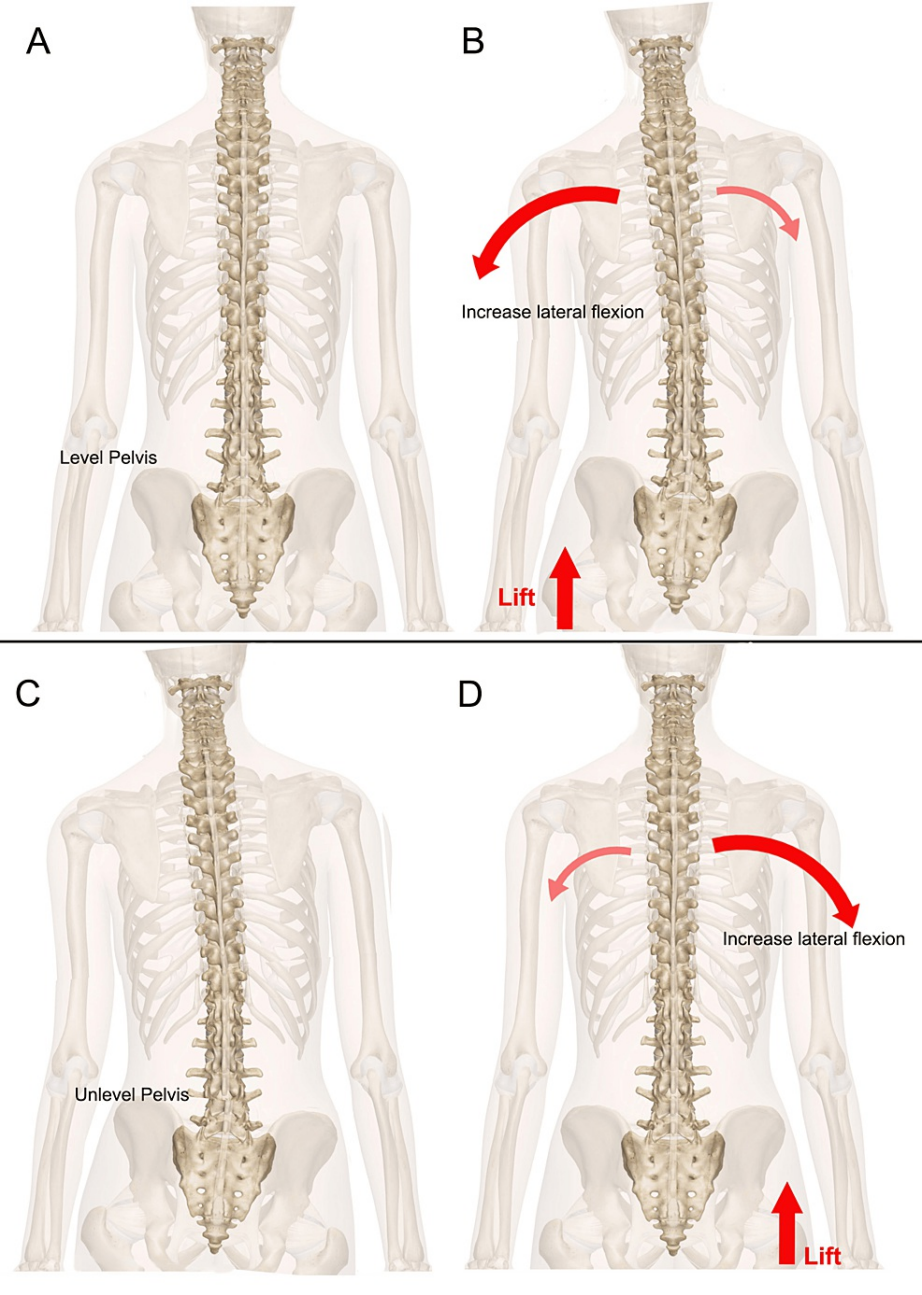
Effects of raising the support in a level and a non-level pelvis. The top row of images (A, B) illustrates schematically the spine's reaction during lateral flexion when a limb is artificially raised with a level pelvis; the bottom row (C, D) of images illustrate the spine's reaction when, however, the pelvis is not level and the elevation compensates for the discrepancy [14]. Image Credit: Author Saverio Colonna

This sort of permanent compensation at pre-skeletal maturity has also been found in patients with pelvic misalignment. Young et al. [14] reported that placing a lift under the foot of a person without pelvic malalignment resulted in greater lumbar lateral flexion toward the side of the now higher iliac crest. In subjects with pelvic malalignment, when the riser was placed under the foot of the lower iliac crest to level the crest, lateral flexion was increased toward the lower iliac crest side. When the body reshapes and adapts to the pelvic misalignment or torsion caused by anatomical LLI, placing a lift under the side of the lower iliac crest effectively raises what the body has adapted to as level. In other words, the unlevel pelvis of those with anatomical LLI has adapted and is now “normal,” and placing a lift under the limb of the side with the lower iliac crest has the same effect as placing a lift under the leg of a level pelvis (Figure 11).

Instrumental measurements

Radiography has long been considered the gold standard for measuring LLIs [29], although radiographic techniques vary and are not without problems. Three predominant methods use radiographs to measure LLIs.

**Figure 12:**
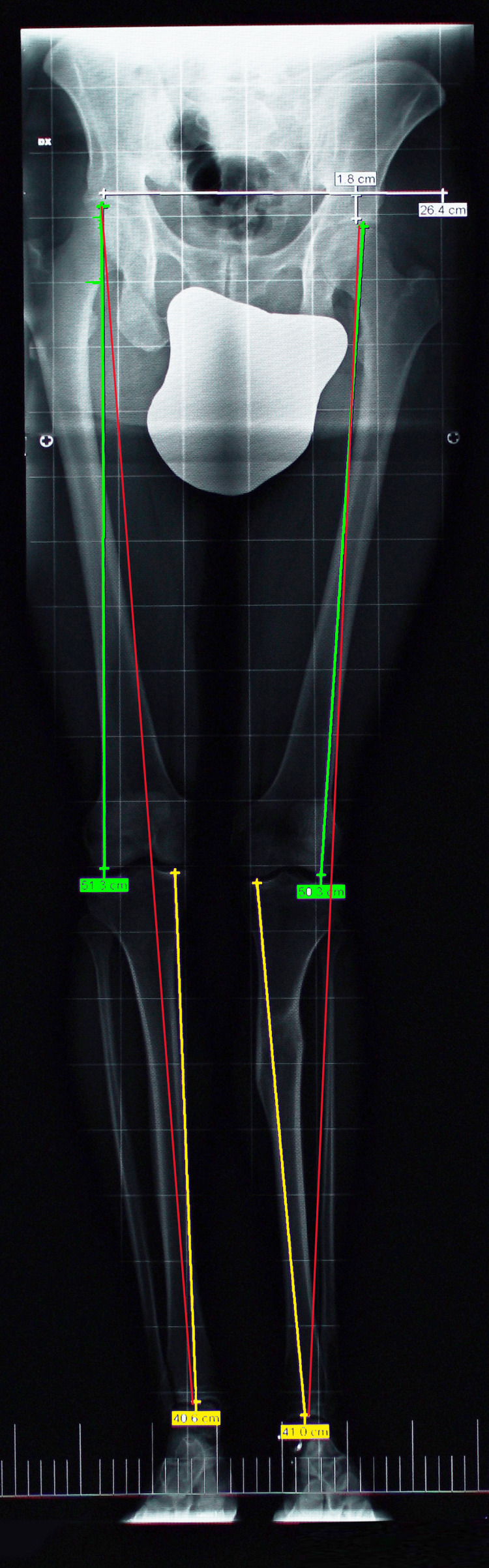
Anteroposterior radiograph in orthostatism of the lower limb (modified teleradiography) performed on X-rays by a computerized system. A patient with LLI due to a displacement fracture of the left tibia that altered the axis and length of the left limb. In addition to evaluating the length discrepancy of the entire limb (including the femur and the tibia), this imaging modality can be used to measure mechanical axis deviations and the angles of knee joint orientation, as proposed by Machen and Stevens [39]. LLI: leg length inequality Image Credit: Author Saverio Colonna

The first is an orthoroentgenogram or teleoroentgenogram (Figure 12), which involves a single exposure of the pelvis and limbs, including the ankles and feet. The advantage of this method is that it requires only one exposure for a global exam of the axis of the lower limbs, allowing the differentiation of sLLIs from fLLIs, but it is subject to distortion due to parallax error [39].

The second method is the scanogram, which has a smaller magnification error than a teleoroentgenogram but requires three radiographic exposures centered on the hip, knee, and ankle joints. The scanogram is performed with the patient in the supine position, is typically associated with increased radiation exposure, does not allow visualization of the entire length of the femur and tibia, and does not consider any shortening related to the foot.

All radiographic techniques measure from some reference point on the proximal femur or pelvis to some reference point on the ankle and do not consider the contribution of the foot to the length of the limb. The third method is computerized digital radiography. This technique minimizes radiation exposure, reduces mathematical error, and is accurate even in the presence of angular deformities [40]. Micro-dose digital radiography is computerized imaging that substantially reduces patients' radiation exposure compared to conventional radiographic techniques. In this method, a relatively old micro-dose digital technique [41], with a more recent development termed EOS (EOS imaging, Paris, France) [42,43], is used to measure limbs. Using a vertical gantry, the patient stands in front of the X-ray unit and remains still during the 20-second scanning process. A continuous series of photon beams collimated to be a point source is projected through the patient to hit a computerized detector. The source assembly and detector move together in a row-by-row motion so that the beam remains horizontal to the patient. Because the detector is efficient at finding and processing the point source of X-ray photons, the patient exposure, during the scan, was six to nine times lower than that of conventional radiographs and four to 23 times lower than that of CT [43].

This almost negligible radiation exposure for the patient makes the technique particularly attractive for problems that require serial radiographic evaluation, such as progressive LLIs. In a study of 25 children with an LLI, Altongy et al. [41] found that micro-dose digital radiography was more accurate than orthoroentgenograms.

Using a device to measure pelvic crest malalignment in standing patients, Petrone et al. [44] found excellent intra- and inter-examiner reliability and validity (ICC = 0.89-0.90) related to anatomical LLI determined by X-ray measurements in asymptomatic subjects. However, the correlation between the pelvic level and the femoral head height was, in a low back pain group, substantially lower [45]. This indicates that some sort of functional pelvic tilt or torsion was present in the low back pain population that was not related to their anatomical LLI. The decreased correlation between a pelvic tilt and an LLI in the back pain group has not been examined in comparison to a functional shorter leg, although the connection between back pain and pelvic torsion is significant.

CT, 3D ultrasound (US), and MRI are also used to determine lower limb length. CT has been proven to have better sensitivity (discrimination ability of approximately 1 mm), offers acceptable reproducibility, and provides limited radiation exposure. Although CT is more accurate and reliable than radiographs, especially when a knee flexion deformity is present [45], it is more expensive. Conventional US has been proven to be inferior to CT for determining leg length, although 3D US has been demonstrated to be an accurate one-step determination of an LLI without ionizing radiation [46]; additionally, its SD for reproducing the limb length measurement is 1.6 mm. MRI has the advantage of having no radiographic risks but has indicated lower reproducibility and accuracy than CT or US [47].

LLI and pathology

The threshold of clinically significant LLI diverges in the literature, and there is currently no international consensus. Length discrepancy in the lower limbs leads to altered posture, gait asymmetry, contracture in knee and hip flexion on the side of the longer limb, contracture of the ankle joint in the plantar flexion position, reduced hip adduction angle, and increased pelvic and trunk external rotation on the side of the shorter lower limb [48-53]. Some scholars have studied the effects of LLIs on a variety of pathological conditions, such as low back pain [54, 55], hip arthritis [56], stress fractures [57], standing balance [58], and running [59].

Clinicians who often find themselves treating young subjects with an LLI, with or without spinal axis alteration, have considerable difficulty in deciding whether to prescribe, together with other therapies such as therapeutic exercise and bracing, compensation for limb discrepancy. The intention that underscores this article is to provide an overview of the literature to offer a more complete picture as well as to impart some final suggestions to those who are faced with the following dilemma: to lift or not to lift.

LLI and scoliosis

Scoliosis is a medical condition characterized by an abnormal curvature of the spine. Scoliosis was defined as a coronal Cobb angle ≥10° [60]. Adolescent idiopathic scoliosis (AIS) and adult scoliosis are two different forms of scoliosis that occur at different stages of life and have distinct characteristics [61].

1. *AIS*

Onset: Typically develops during adolescence, usually between the ages of 10 and 18, with the majority of cases diagnosed during puberty growth spurts.

Cause: The term "idiopathic" means that the cause is unknown. AIS occurs without any apparent underlying reason, such as neuromuscular disorders or congenital abnormalities.

Progression: The curvature of the spine can progress during the rapid growth period of adolescence, especially during puberty. The rate of progression varies among individuals.

Treatment: Treatment options for AIS depend on the severity of the curvature and may include observation, bracing, or surgery. Bracing is often recommended for moderate curves to prevent progression, while surgery may be necessary for severe cases to correct the curvature and stabilize the spine.

2. *Adult Scoliosis*

Onset: Adult scoliosis can develop for the first time in adulthood or may result from the progression of AIS into adulthood.

Cause: Adult scoliosis can have various causes, including degenerative changes in the spine due to aging (degenerative scoliosis), progression of untreated AIS, or secondary to conditions such as osteoporosis, spinal stenosis, or prior spinal surgery.

Progression: While scoliosis in adults may progress, it typically progresses at a slower rate compared to adolescent idiopathic scoliosis. Progression can be influenced by factors such as aging, degenerative changes, and other underlying health conditions.

It is a common experience, and several scholars [61-64] have demonstrated that AIS is frequently associated with a significant prevalence of lower limb biomechanical anomalies, including pelvic height differences and LLIs. However, not all authors agree [60,65,66], which is why we performed a literature review.

Search strategy

The literature search was conducted in PubMed, Google Scholar, Cochrane Library, and Scopus databases. The search was conducted using keywords for PubMed: ‘Leg Length Inequality"[Mesh] OR "Leg Length Discrepancies"[Mesh] OR "Leg Length Difference"[Mesh] OR "Leg Length"[Mesh]) AND ("Scoliosis"[Mesh] OR "Spinal Curvatures"[Mesh]); and for Google Scholar, Cochrane Library, and Scopus databases: Leg Length Inequality" OR "Leg Length Discrepancies" OR "Leg Length Difference"OR "Leg Length"AND ("Scoliosis"OR "Spinal Curvatures").

Eligibility criteria

All studies addressing LLI or LLD or leg length difference and scoliosis or spinal curvatures, having an association, link, or relationship with scoliosis or spinal curvatures, and published from 1980 to 2024 were included.

Sample size

The result in PubMed was 99 works, 11 of which were research-related; in Google Scholar, it was 862 results, of which 25 were related to the search; and in Cochrane Library and Scopus, there were 0 results. Of these 36 studies, 18 studies were eliminated, 16 due to irrelevant results or outcomes, and two were not available for full access, therefore the final sample size for the review came out to be 18, the details of which are given in the results section (Table 1).

**Table 1:** Studies included in the review and their schematized outcomes. Literature review of correlations between LLI and scoliosis LBP: low back pain; AIS: adolescent idiopathic scoliosis; LLI: leg length inequality

No.	Author/year of study	Objective	Study type	Sample size/age (years)	Size of LLI	Association	Results
1.	Papaionnu et al. 2018 [38]	Evaluate LBP and its association with high heels	Retrospective observational	N: 23, range 17-39 years. The discrepancy had been present through a large part of the patients’ childhoods	Range: 1.2-5.25 cm	Yes	The Cobb angle varied in proportion to the severity of anisomelia. The lateral curve of the spine, convex toward the short limb, was seen in all patients and the first sacral segment was the lowest vertebra
2.	Gibson et al. 1983 [37]	Assess structural changes in the spine following leg-length inequality developing after skeletal maturity, and assess the effect upon the spine of correcting the inequality	Cross-sectional	N: 50. The patients investigated had acquired LLI after skeletal maturity	Average: 3 cm, range: 1.5 to 5.5 cm	Yes	The lumbar scoliosis associated with the LLI was compensatory: after equalization of leg length, the overall curve and the axial rotation were corrected completely. There was also an equal range of lateral flexion to either side after correction
3.	Hoikka et al. 1989 [65]	Analyze the correlations between LLI, pelvic tilt, and lumbar scoliosis in patients with chronic LBP	Cross-sectional	N: 100, mean: 40 years	Mean: 5 mm	No	The LLI correlated well with the iliac crest tilt, moderately with the sacral tilt, and poorly with the lumbar scoliosis
4.	Irvin 1991 [67]	The relationship between LLI, non-levelness of the sacral base, and scoliosis	Prospective	N: 42 adults	Unclear	Yes	Lumbar scoliosis < 20° is reduced after leveling the sacral base by wearing a lift of graduated thickness inside their shorter leg shoe
5.	Zabjek et al. 2001 [68]	Acute spinal and three-dimensional postural adaptations induced by a shoe lift in a population of idiopathic scoliosis	Prospective	N: 46, mean: 12 years	For patients with a pelvic obliquity, with or without a leg length inequality, a shoe lift was placed under the side of the obliquity	Yes	The implementation of a shoe lift independent of the type of curve and amplitude significantly decreased the Cobb angle
6.	Raczkowski et al. 2010 [35]	Assess the possibility of correction of spine functional curvature and potential real equalization of LLI	Retrospective observational	N: 369, range 5-17 years	Among 369 children, a discrepancy of 0.5 cm was observed in 27 children, 1 cm in 329, 1.5 cm in 9, and 2 cm in 4 children	Yes	Leg length discrepancy equalization results in the elimination of scoliosis. The time needed for real equalization of the discrepancy was 11.3 months
7.	Betsch et al. 2013 [69]	Simulated LLIs to find the point at which such discrepancies do in fact alter spinal posture.	Cross-sectional	N: 100, mean 34 years	Up to 60 mm	Yes	LLIs of more than 20 mm lead to significant changes in the spinal posture: significant correlation between LLIs and pelvic position
8.	Landauer 2013 [70]	The purpose was to diagnose the causes of LLI and review their surgical treatment options.	Unclear	N: 15 cases of 246 patients	Unclear	Yes	With the correct treatment of these cases, a partial correction of the scoliosis happens automatically. This study provides an initial overview of LLI and recommends to take the lower extremities into account when diagnosing and treating scoliosis
9.	Cho et al. 2017 [71]	The purpose was to assess the association between sacral slanting and adjacent structures in patients with AIS	Cross-sectional	N: 313; mean: 15.3 years	The percentages of patients with LLI of more than 10 mm 10.6%, 8 mm 16.8%, and 6 mm were 27.8%	Yes	LLI is correlated with pelvic obliquity and lumbar curve, but not with sacral slanting
10.	Sekiya et al. 2018 [42]	Evaluate the differences between functional and structural LLIs and determine whether there are true LLIs in patients with AIS	Cross-sectional	N: 82, range: 10-18 years	No patients had a structural LLI ≥ 10 mm, although 18 patients had a functional LLI ≥ 10 mm	Yes, but only functional LLI	Functional LLIs were significantly larger than structural; functional and structural LLIs were significantly correlated with pelvic obliquity; functional LLI, but not structural LLI, was significantly correlated with the lumbar but not thoracic Cobb angle
11.	Grivas et al. 2018 [66]	Mild limb length inequality aiming to investigate its impact on pelvic imbalance, spinal posture, and scoliotic curve, using surface topography analysis	Prospective	N: 20, range 9-15 years		No	We recommend the correction of the mild LLI below the classic definition of 2.0 cm with the use of shoe elevations, as the parameters measured were statistically significantly changed and influenced pelvic imbalance and spinal posture
12.	Ploumis et al. 2018 [60]	It aimed to define if there is a quantitative association between pelvic obliquity, LLI, and the scoliotic curve in an adolescent pediatric population initially presenting for scoliosis evaluation in a pediatric clinic and to evaluate the progression of the scoliotic curve in relation to the different amounts of LLI	Retrospective	N: 73, range: 10-16 years for girls and 10-18 years for boys		No	LLI is uncommon in adolescents with idiopathic thoracic or thoracolumbar scoliosis. Unlike the patients with smaller anisomelia, the patients who had LLI of > 10 mm showed always pelvic obliquity and major thoracic or thoracolumbar scoliotic curves. Even though LLI remained stable after at least two years of growth, scoliosis and iliac difference progressed despite treatment
13.	Pinto et al. 2019 [72]	The objective of this study was to evaluate LLI in adolescent idiopathic scoliosis	Retrospective	N: 80, mean: 12.4 years	Mean value of 0.52 cm; 84.4% less than 1 cm, 14.3% of 1-1.5 cm, and only one patient had more than 1.5 cm	Yes	In a population with AIS and small LLI values, the extent of the shortening has a stronger impact on the location than on the dimension of the scoliotic curvature. A more thorough study on the importance of LLI <1 cm in the development of biomechanical changes in the spine would therefore be of great interest
14.	Banno et al. 2020 [73]	Assessed the prevalence of pelvic obliquity according to the Lenke classification and investigated its influence on coronal alignment according to radiographic parameters by comparing patients with and without pelvic obliquity	Retrospective	N: 131, mean: 14.2 years	No patients had a structural leg discrepancy ≥ 10 mm; six patients had a functional leg length discrepancy ≥ 10 mm,	Yes	In the patients with functional LLI, the pelvic coronal obliquity angle and iliac crest height differences were correlated with the femoral head height differences
15.	Kobayashi et al. 2020 [64]	Examine the correlations between scoliosis and the cause and severity of LLI and pelvic obliquity.	Cross-sectional	N: 25, range 5-18 years	Range: 27–65 mm	Yes	The Cobb angle was significantly related to the severity of the LLI, more severe scoliosis occurred at an LLI ≥30 mm.
16.	Buyukaslan et al. 2022 [25]	Clinical and radiological features of functional scoliosis due to LLI and LLI concurrent with AIS	Retrospective	N: 47, range: 10-18 years	12.3 ± 9.2 mm	Yes	LLI may develop as a result of adaptive changes due to scoliosis, or a concurrent condition to scoliosis. LLI was compensated by functional scoliosis, with a lumbar convexity to the short leg side in most cases in the LLI group
17.	Hamada et al. 2022 [74]	Correlations between LLI and lateral curvature of the lumbar spine using standing radiographs	Retrospective	N: 113, range 10- 65 years		Yes	Lumbar Cobb angle tends to be >10° if the difference in leg lengths is >20 mm
18.	Marsiolo et al. 2023 [75]	Research the presence of vertebral rotation in functional scoliosis caused by LLI	Retrospective	N: 89 <16 years		Yes	Functional scoliosis secondary to LLI can cause vertebral rotation, and 5 mm of LLI can cause sacral shelf inclination and rotation

In some cases, LLI can be secondary to scoliosis, especially with compensatory scoliosis. In these cases, LLI appears as the result of an asymmetrical load on the lower limbs [14,68]. Just as LLI is either functional or structural [13,76], scoliosis is also divided into functional and structural cases, each of which has different biomechanical characteristics. Rothschild et al. [77] posited that while structural scoliosis manifests with a noticeable hump in Adam's flexion test, a common method for assessing scoliosis, functional scoliosis does not exhibit this characteristic hump during trunk flexion.

LLI causes pelvic obliquity, precisely the non-horizontality of the axis that passes through the tip of the superior iliac crest, in the frontal plane [14,23,50,52,68,78] and an inclination of the sacral base [25], which is defined as sacral slanting [79] or sacral shelf inclination [75]. A rather rigid junction of the L5 vertebra with the sacral bone is often observed in lumbar scoliosis, with convexity directed toward the shorter leg [35]. Additionally, as supported by some authors [80], a short left limb, for example, involves a pelvis twisted or inclined to the left (on the lower side), left sacral inclination, the lumbar curve is convex to the left if there is a single curve; if the curve is double, then the counter cranial curve, defined as a secondary curve, is on the right.

In patients with single or double degenerative scoliotic curves, statistically significant differences are found in the position of the apex of the curve (laterality) compared to the side of the pelvic slope: the highest iliac crest is significantly more present on the curve's concave side in patients with a single curve (79%) compared to those with a double curve (48%). Patients with double curves (72%) are significantly more likely than patients with single curves (49%) to have the lower iliac crest on the same coronal side as the lumbar concavity [80].

Cho et al. [71], analyzing the X-rays of 303 patients with scoliosis, reported a positive correlation between lumbar curve, sacral inclination, and pelvic inclination; pelvic obliquity was correlated with LLI; however, a partial correlation analysis revealed that LLI was not directly correlated with sacral slanting. The authors concluded that it was not possible to determine the exact cause-and-effect relationship between these parameters.

Schwender and Denis [81] studied 50 AIS patients with a left lumbar curve greater than 40° to determine whether the lumbosacral hemicurve predisposed these patients to coronal decompensation. They found that the sacrum, which is a component of the hemicurve, was tilted toward the lumbar curve in 90% of cases, and 63% of patients also had an iliac tilt. For patients with primary lumbar or thoracolumbar curves, 100% had sacral and iliac tilt toward the lumbar curve. Schwender and Denis [81] proposed, therefore, that sacral inclination (whether sacral slanting or sacral shelf) could be a compensatory mechanism for lumbar curves, which would contribute to LLI through its connection to the pelvis. The results of a study [82] that reported a reduction in the height difference of the lower limb after spinal surgery could align with this perspective of the relationships between the different alterations that scoliosis entails; notably, this phenomenon only manifested itself in patients who initially had a shorter left limb. Several other scholars have defined LLI by the discrepancy in the femoral head's position on pelvic radiographs [54,60,83].

We share the position of Machen and Stevens [39] and Sabharwal [84], who argued that comprehensive X-rays of the lower extremities performed with the patient in an upright position are a superior diagnostic screening tool for the condition in question. Both LLI and scoliosis are caused by differences in bone length and abnormal angles in the bones. Standing X-rays allow a view of the patient's bones in the upright position, which is the posture that displays such irregularities most prominently. This method, however, cannot reveal whether the LLI is structural or functional. Other researchers have concurrently considered both the pelvis and spine as a whole and the lower limbs globally, most recently Sekiya et al. [42] and Hamada et al. [74].

Regarding structural scoliosis and its relationship with the pelvic arrangement and limb length, Sekiya et al. [42] evaluated 82 adolescents (age range, 10-18 years) with idiopathic scoliosis using the biplanar low-dose X-ray imaging device system (EOS), to evaluate the type of LLI, concluding that sLLIs were less common than fLLIs. Of the cohort, none had an sLLI greater than 10 mm, while 18 had a fLLI greater than 10 mm. Both fLLIs and sLLIs are related to the inclination of the sacrum; furthermore, only fLLIs appear to correlate with lumbar Cobb angles (the radiographic angle that commonly quantifies the severity of the scoliotic curve), although neither type of LLI appears to correlate with thoracic Cobb angles. The authors suggest that fLLIs may be compensatory for lumbar curves, but the mechanisms involved have not been studied.

The reason why LLIs are related to lumbar curves but not to thoracic curves can be understood from the results of a recent study [85] in which scholars found that specific types of scoliosis (Type 1 AR and 1AL of Lenke) that involve the dorsal spine correlate with the asymmetry of the articular facets (zygapophysis) of the L3-L4 and L4-L5 vertebrae. The facet joints between L4 and L5 are most involved in the structural asymmetry of the lumbar spine, which can lead to both scoliotic alterations and other spinal pathologies [86]. It follows, therefore, that an LLI has a greater relationship with the lumbar curve than the thoracic curve, which is often compensatory in nature, just as an asymmetry of the lumbar facets is related to a thoracic curve.

The belief that there may be anatomical asymmetries of the lower limbs is widely shared, but fewer scholars accept the idea that other anatomical structures, such as the sacroiliac structures and the facet joints, may also have asymmetries of anatomical conformation.

Sometimes there may be structural alterations even at two or more levels in the opposite or ipsilateral direction. It would be interesting, when carrying out scoliosis assessments, to identify the tilts of the vertebrae involved in scoliosis and, taking into consideration the one with the greatest degree of tilt, to establish whether it is of a functional or structural nature. Obviously, to do this, it would be necessary to have, in addition to the orthoroentgenogram, also MRI or CT scan evaluation of the section under examination, which is able to identify an anatomical alteration of the surfaces of the articular facets.

A more recent study [74], in which scientists used a heterogeneously aged sample (10-65 years) and full-length standing anteroposterior radiographs of the spine and lower limbs, also demonstrates a strong positive correlation between the degree of LLI and the lumbar Cobb angle; furthermore, using a derived regression equation, estimates a lumbar Cobb angle of approximately 10° at a LLI of 20 mm, which represents the pathological cut-off for lumbar scoliosis adopted in several other studies [60,87]. The authors, aligned with previous findings from other authors [38,88], suggest that an LLI ≥ 20 mm should be the threshold for recommending corrective surgical treatment. However, the degree of LLI sufficient to justify surgical correction remains undetermined because while some authors state that even differences of ≤ 10 mm [57,83] have implications, others maintain that only differences of ≥ 30 mm [89,90] are potentially harmful.

Relationship between LLI and pelvic inclination in scoliosis patients

By definition, a functional curve resolves, and the spine resumes a straight configuration when the patient lies down or tilts. Rothschild et al. [77] found that functional scoliosis in adolescent patients (AIS) does not present with a hump in Adam's flexion test, unlike structural scoliosis. Lateral curvature of the spine that can be completely corrected by using a riser to balance an LLI is an example of functional scoliosis [91]. The opinion has prevailed that the asymmetry of the lower limbs determines a pelvic inclination, which can induce functional scoliosis and is therefore reversible and negative on Adam's test; however, scholars are not unanimous on this. Hoikka et al. [65] in a study of 100 patients with an average LLI of 5 mm and an average age of 47 years, found a correlation between LLI and sacral inclination but no relationship between LLI and lumbar Cobb degrees.

The conclusions of a recent study [75], in which researchers examined 89 X-rays of scoliotic subjects with a Cobb angle of less than 15°, are that functional scoliosis due to an LLI can lead to vertebral rotation, with a direct correlation between the LLI, sacral base inclination, and vertebral rotation. Additionally, an inequality in limb length greater than 5 mm is the threshold value beyond which the sacral base could tilt, causing rotation of the spine. These findings cast doubt on whether LLIs result in reversible scoliosis without hump formation during Adam's test [75].

The results presented in Papaioannou et al.'s [38] study, which included only young adult patients whose LLI ranged from 1.2 cm to 5.2 cm, underscore that LLIs present since childhood align with the results of previous studies [37]; in fact, they suggested that a long period of functional scoliosis can cause permanent biomechanical changes in the lumbar spine. The period for which the spine is subjected to functional scoliosis also seems to affect the risk of degenerative changes. Manganiello et al. conducted two studies [92, 93] to analyze the impact of LLI on the lumbar spine and also suggested that a small LLI may induce a more significant malalignment of the lumbar region than a larger LLI (> 2 cm). They also proposed that an LLI may be the prime cause of the onset of structured scoliosis. Furthermore, other scholars have suggested that a severe LLI can permanently modify the biomechanics of the lumbar spine in the long term, potentially increasing susceptibility to irremediable scoliosis [37,94].

An LLI, by altering the support of the acetabulum on the femoral head, alters the balance of the pelvis. Several scholars have reported that LLIs are related to pelvic tilt in the coronal plane, which is also called pelvic obliquity [11,42,57,66,71,95-97]. In a sample of 524 patients with degenerative scoliosis and an average age of 60.7 years, Radcliff et al. [80] reported that the prevalence of pelvic obliquity in patients with a single degenerative scoliotic curve was 91%. In this group, the apex of scoliosis was on the same side of the lower iliac crest in 79% of patients; in the remaining part of the group with a double scoliotic curve, the apex of scoliosis was on the same side of the lower iliac crest only in 48% of patients.

**Figure 13:**
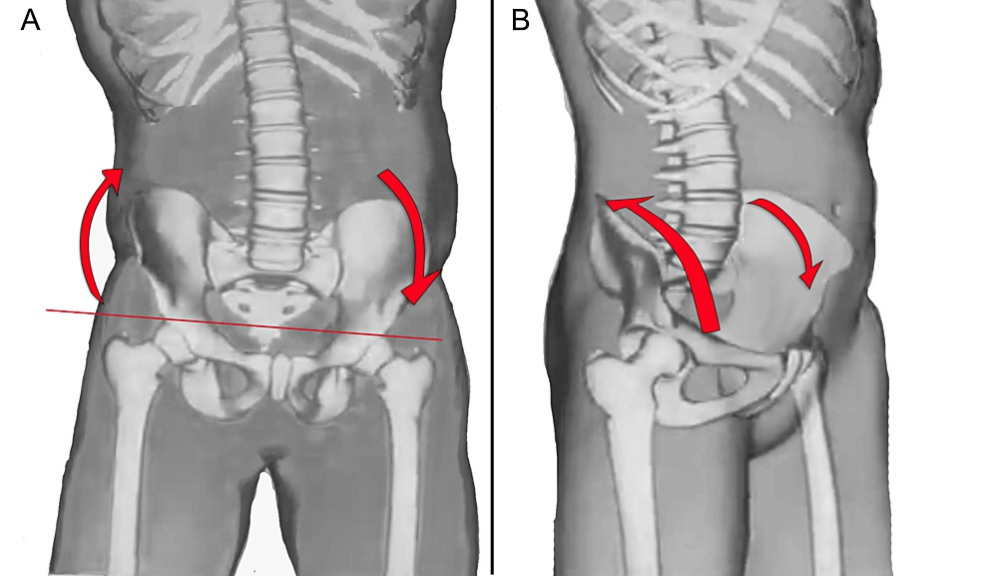
The images schematically illustrate the adaptation of the pelvis in artificially induced LLI situations. Artificial elevation of the right sole raises the ipsilateral pelvis and induces functional left lumbar scoliosis in the coronal plane (A). In the sagittal plane, lifting the sole of the right foot, in most cases, causes posterior iliac rotation ipsilaterally and anterior iliac rotation contralaterally (B) [76]. LLI: leg length inequality Image Credit: Author Saverio Colonna

Some scholars have studied the immediate effects of artificially induced LLI by using different measurement techniques and have found that LLIs affect pelvic placement by creating pelvic obliquity. Different sizes of platform lifts have been used to artificially induce an LLI in healthy participants [11], including adolescents [66] and a group of soldiers [57], to determine their impact on pelvic placement. The results suggested that increasing the height of the foot's contact with the ground, which elevates the ipsilateral pelvis, causes pelvic obliquity, as illustrated in Figure 13.

A few scholars have studied the effects of an LLI on pelvic disposition in the transverse plane or pelvic rotation [53,66,95]. With an artificially induced or subsequent anatomical condition of LLI, scientists have doubts about the sagittal and transverse plane adaptations even when the pelvis behaves clearly in the coronal plane adaptation. Some scholars have suggested that the two innominate bones do not move in tandem in the compensatory pelvic tilt. Beaudoin et al. [95] used a motion analysis system in 20 healthy female participants to quantify the acute 3D postural changes to the pelvis, trunk, scapular belt, and head induced artificially by a shoe lift, finding asymmetrical versions of the right and left iliac bones in the presence of a shoe lift with a posterior rotation of the innominate bone on the side of the shoe lift becoming longer and an anterior rotation on the side of the leg becoming shorter. Grivas et al. [66], using formetric 4D, reported that an artificially induced LLI was significantly correlated to pelvic rotation; nevertheless, the compensatory strategy used was not described.

**Figure 14:**
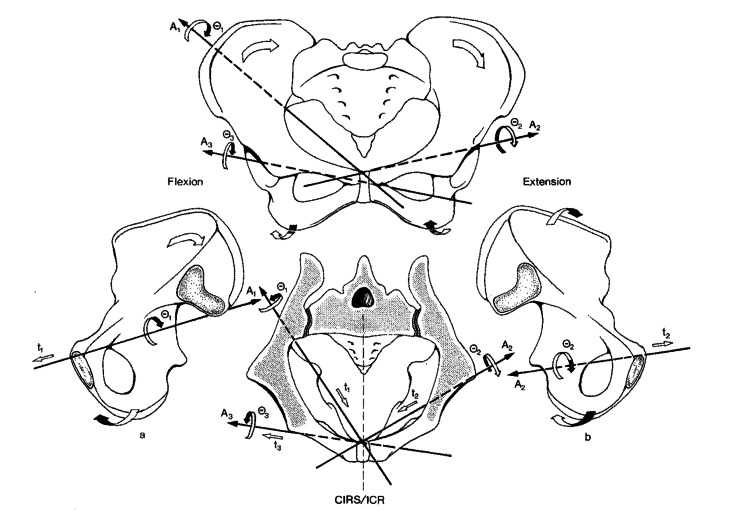
Schematization of the iliac bones' movements proposed by Lavignolle et al. Asymmetrical pelvic movements. Zone A1 houses the axes of movement of the right iliac bone with respect to the sacrum (right sacroiliac joint). Zone A2 contains the axes of movement of the left iliac bone with respect to the sacrum (left sacroiliac joint). Zone A3 includes the axes of movement of the right iliac bone with respect to the left iliac bone. Areas t1, t2, and t3 present translations along axes A1, A2, and A3, respectively. Areas 01, 02, and 03 present rotations around axes A1, A2, and A3, respectively. ICR: instantaneous centers of rotation in the sagittal plane (the intersection of axes A1,  A2, and A3 with the median sagittal plane). Image Credit: Lavignolle et al., 1983 [104]; used with permission

These results confirm the findings of other studies [11,98,99], although some older studies [100,101] have presented contradictory results: the iliac bone tended to rotate anteriorly on the side of the longer leg and posteriorly on the side of the shorter leg. A possible response to these contradictory perspectives is that when the pelvis compensates, the sagittal, coronal, and transverse planes do not move as distinctly as their theoretical divisions imply. The model of posterior/anterior rotation in the sagittal plane, as proposed by Don Tigny [102], is useful for teaching purposes but does not correspond to reality. The first study, to our knowledge, that reiterated this concept was by Pitkin and Pheasant (1936) [103], who stated that only the flexion-extension movement of the trunk, which occurs in isolation in the sagittal plane and symmetrically involves both iliac bones, appeared to occur mainly at the level of the coxofemoral joints, and to a lesser extent, at the sacroiliac level; all the other movements occurred on an oblique axis that joined the two fixed points of the pelvis, which are the sacroiliac joint and the pubic symphysis. Pitkin and Pheasant [103] posited that the differential movement that occurs between the two iliac muscles created a pelvic twist that physiologically reached approximately 11° and could reach 14.6° in pathological subjects. If this amount of movement occurred only in the sagittal plane, then it would be dangerous because it would lead to a linear movement of more than 2 cm at the level of the pubis with consequent dislocation of the symphysis. More recently, other authors [104] have reported data on the in-vivo movement of the axes of the pelvis, which confirmed that inter-iliac horizontal axes do not exist but oblique axes do exist (Figure 14).

**Figure 15:**
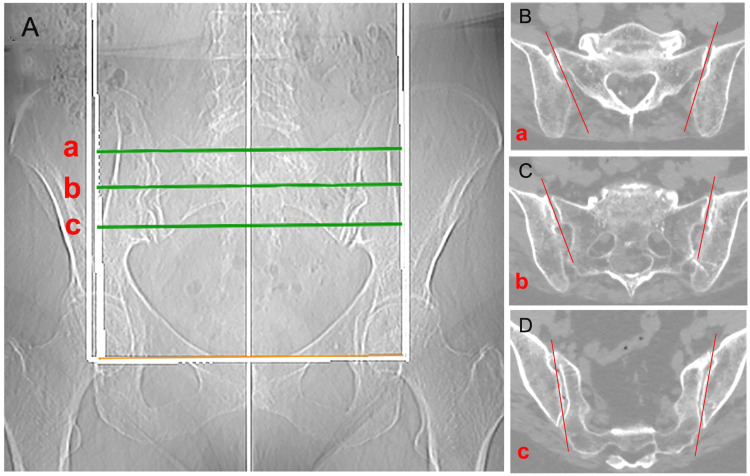
CT scan of the sacroiliac joints. In the coronal view (A), the green lines show in the axial cuts; a) upper (B); b) middle (C); and c) lower (D) portions. The red lines highlight obliquities of the axes of the articular surfaces. Image Credit: Author Saverio Colonna

Further proof that differentiated movement does not exist between the two iliac bones, which would indicate a pelvic twist isolated to the sagittal plane, is the anatomical conformation of the surfaces of the sacroiliac bone: the axis representing the interline at any level is not on the sagittal but on an oblique plane (Figure 15).

It should, therefore, be restated that the body attempts to compensate for any structural abnormalities in symmetry by replacing anteversion and retroversion, movements that are performed strictly in the sagittal plane, by opening (opening the large pelvis and closing the small pelvis) and closing the iliacus (closing the large pelvis and opening the small pelvis), as proposed in some works [105,106].

**Figure 16:**
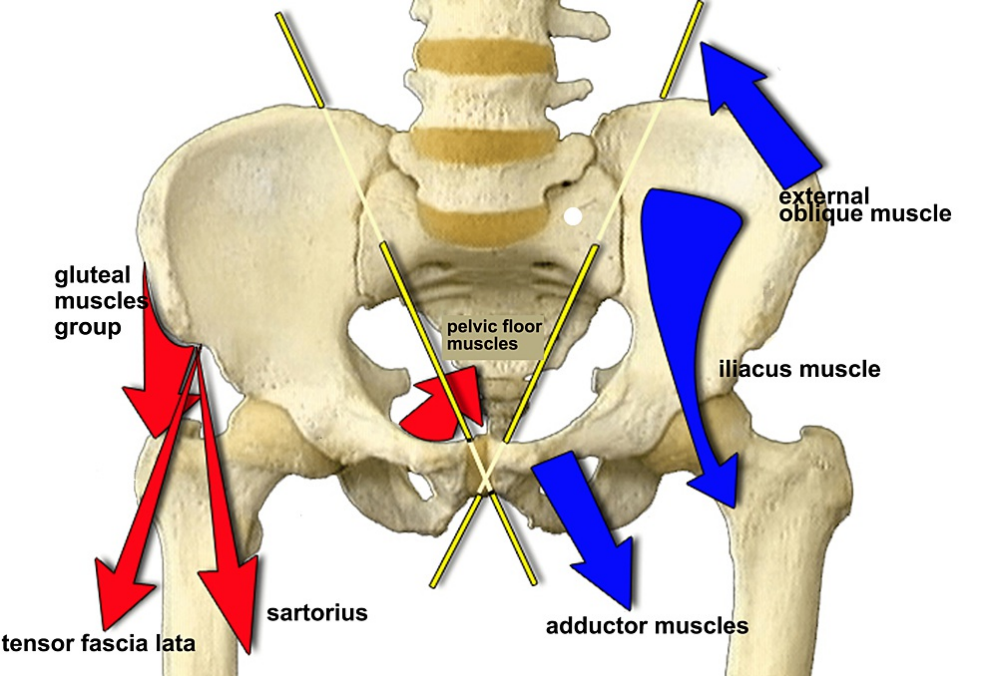
Schematization of the pelvis with oblique axes. Schematic illustration of the oblique axis in which the twisting movements of the pelvis and the related muscles that determine said movements are typically performed, as proposed by Colonna [105]. Image Credit: Author Saverio Colonna

**Figure 17:**
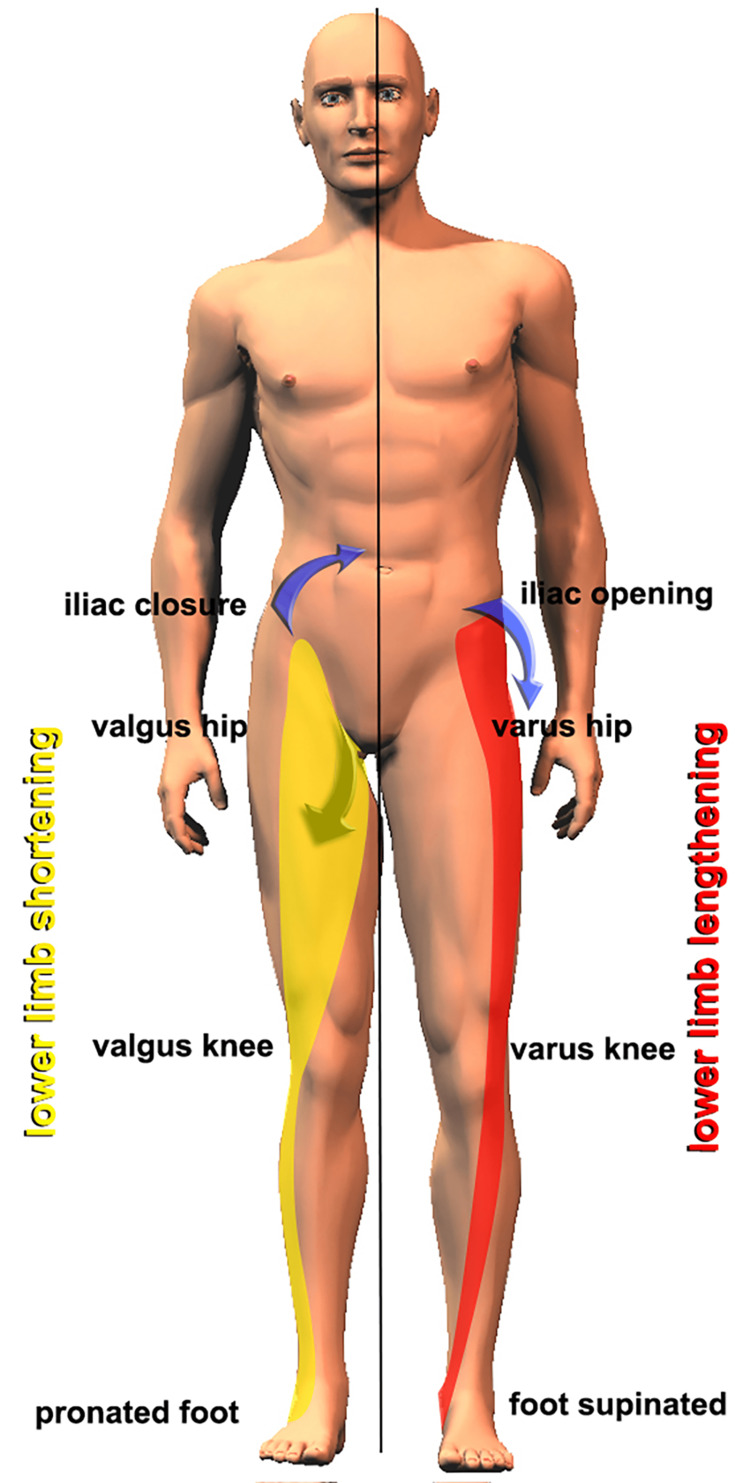
Myofascial chains that manage pelvic twists on the oblique axis. Schematization of the functional changes in the pelvis and lower limbs produced by the hyperactivation of the closing myofascial chain (on the left in yellow) and the opening myofascial chain (on the right in red). Image Credit: Colonna, 2007 [105]; used with permission

When analyzing the opening and closing movements of the ilia, it should be underscored that much depends on the inclination of the oblique axis, which depends on the dimensions of the sacrum and the iliac bone. The wider the sacrum, as in the female sex, the more the plane of movement advances toward the sagittal plane, whereas the narrower the pelvis and sacrum, typical of the male sex, the more the plane of movement advances toward the coronal plane. When assessing pelvic torsion, if the ilia positions are used and evaluated through the relative position of the anterosuperior and posterosuperior iliac spines, then discordant responses are likely because of the different conformation of the pelvis. Colonna [105] reported which muscles determined the opening and closing of the iliac bone (Figure 16), which myofascial chain systems intervened to determine the iliac opening with consequent functional lengthening of the lower limb, and which, conversely, determined closure with relative functional shortening (Figure 17). Using these in data, not in isolation, but together with the previously exposed assessments, can be a useful additional element to understand what type of LLI we are dealing with. For intellectual honesty, we must add that myofascial chains are not supported by firm data in the literature. It is a theory that requires further investigation.

An artificially induced LLI leads to an increase in pelvic tilt and pelvic torsion, but it should be noted that the changes occur non-proportionally, as only about half of the simulated lift is transferred to the pelvic tilt, and even less of the lift affects torsion [107]. These findings were first described by Drerup et al. [108], who stated that the effects of different LLIs were difficult to predict due to the complex interactions in the kinematic chain of the lower limbs and pelvis. The different simulated leg lengths may not be transferred directly to the pelvis. One explanation is that a twist could occur in the sacroiliac joints, an anatomical asymmetry of the pelvis may exist, or the suprapelvic muscles may be hypertonic [13,109].

A recent systematic review of 40 articles on the use of insoles in idiopathic scoliosis [77] reported the following.

“It is clear from the studies that the prescription of customized insoles and orthoses depends on a series of factors; foot lifting seems to be indicated in the presence of functional lumbar scoliosis on the short limb side [35, 38, 57, 67, 78, 110] and, in line with the findings of Juhl et al. [111], we believe that it should only be applied when the level of the sacrum is parallel to that of the femoral heads.”

**Figure 18:**
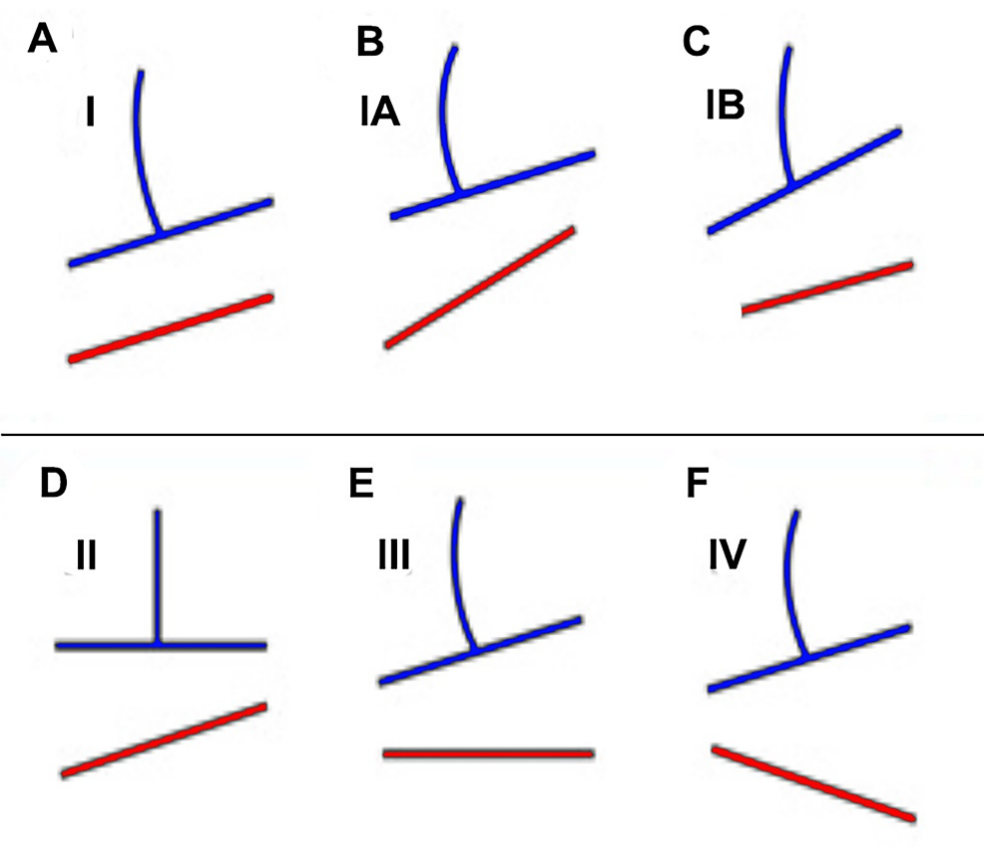
Schematic examples of pelvic height differences proposed by the Lloyd and Eimerbrink classification. Type I (A): The levels of the sacrum (blue) and the femoral heads (red) are parallel; Type IA (B): the flatness of the sacrum and hips is on the same side as the femoral head misalignment, which is more accentuated; Type IB (C): the flatness of the hips and the sacrum is ipsilateral but has greater obliquity of the sacral base compared to the femoral heads; Type II (D): the sacrum is level, but the femoral heads are not; Type III (E): the femoral heads are level, but the sacrum is not; Type IV (F): the inclination of the sacrum and femoral heads are on opposite sides. Adapted from [112].

**Figure 19:**
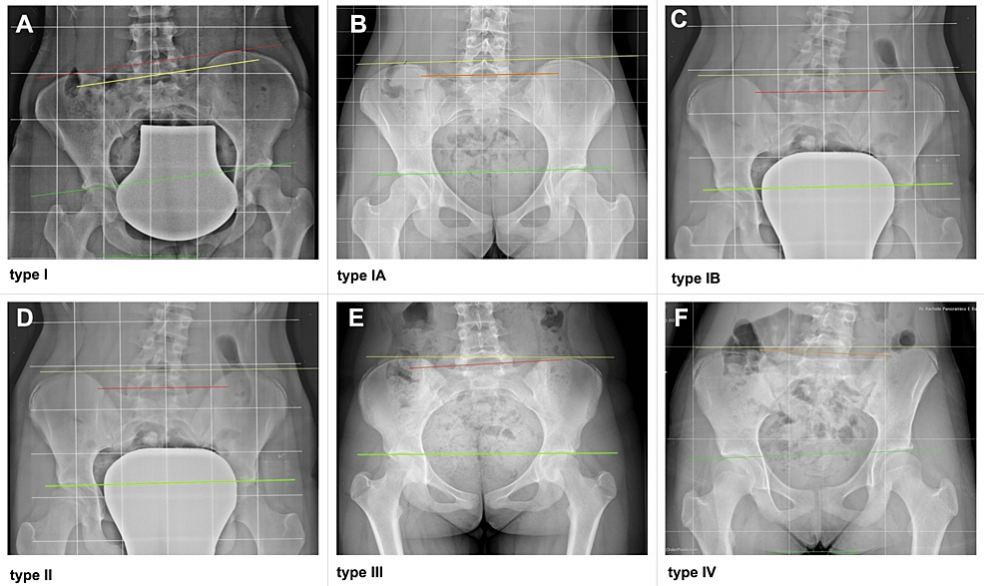
Examples of pelvic asymmetries, as proposed by Lloyd and Eimerbrink. Type I: The levels of the sacrum (red line) and femoral heads (green line) are parallel (A); Type IA: the planarity of the sacrum and the heads is on the same side with the femoral head misalignment, which is more accentuated (B); Type IB: the planarity of the sacrum and hips is on the same side but with greater obliquity of the sacral base compared to the femoral heads (C); Type II: the sacrum is level, but the femoral heads are not (D); (e) Type III: the femoral heads are level, but the sacrum is not (E); (f) Type IV: inclination of the sacrum and femoral heads are on opposite sides (F). Image Credit: Author Saverio Colonna

In fact, Heiling [112], using Lloyd and Eimerbrink's classification, reported that there may be different types of relationships between the axes that connect the iliac crests, the sacral bases (sacral slanting or sacral shelf), and the femoral heads (Figures 18, 19).

Juhl et al. [111], studying 421 patients with low back pain, reported that the majority had a significant LLI, and the majority of these had a shorter leg on the dominant hand side. Using a cut-off of 4 mm for LLI, the most common pattern of pelvic postural asymmetry was Type I (27.8%); the rarest was Type IV. When the LLI limit increased to 10 mm and 15 mm, the Type IB model predominated, and the Type II model died out. As the cut-off for LLI increased, the frequency of sacral height differences increased, with functional scoliosis tending to be convex with respect to the lower side of the sacrum. There was no age-dependent progression from one pelvic type to another. The frequency of sacral dysfunction, represented by the functionally similar Types IB and III groups combined, remained constant across the age spectrum.

In patients with a Type IV pelvic postural pattern, scoliosis tended to follow the sacral difference. A disproportionately large number of patients in the left-handed group had a Type IV pelvic pattern. In the presence of idiopathic scoliosis, it is essential to verify whether an anatomically shorter limb actually exists, as fLLI are more frequent [42]. Using insoles in the absence of an sLLI can induce a compensatory lumbar or lumbosacral curve [61]. The authors conclude that footlifting seems to be indicated in the presence of certain types of functional scoliosis [61].

For scoliosis, a scoliometer is simple tool to verify the usefulness of compensating for an LLI (see Figure 7). Additionally, a scoliometer can be used to check whether a rise positioned under the foot of the shorter limb reduces the asymmetry of the pelvis [25] and the degree of the lumbar hump.

A new evaluation proposal

**Figure 20:**
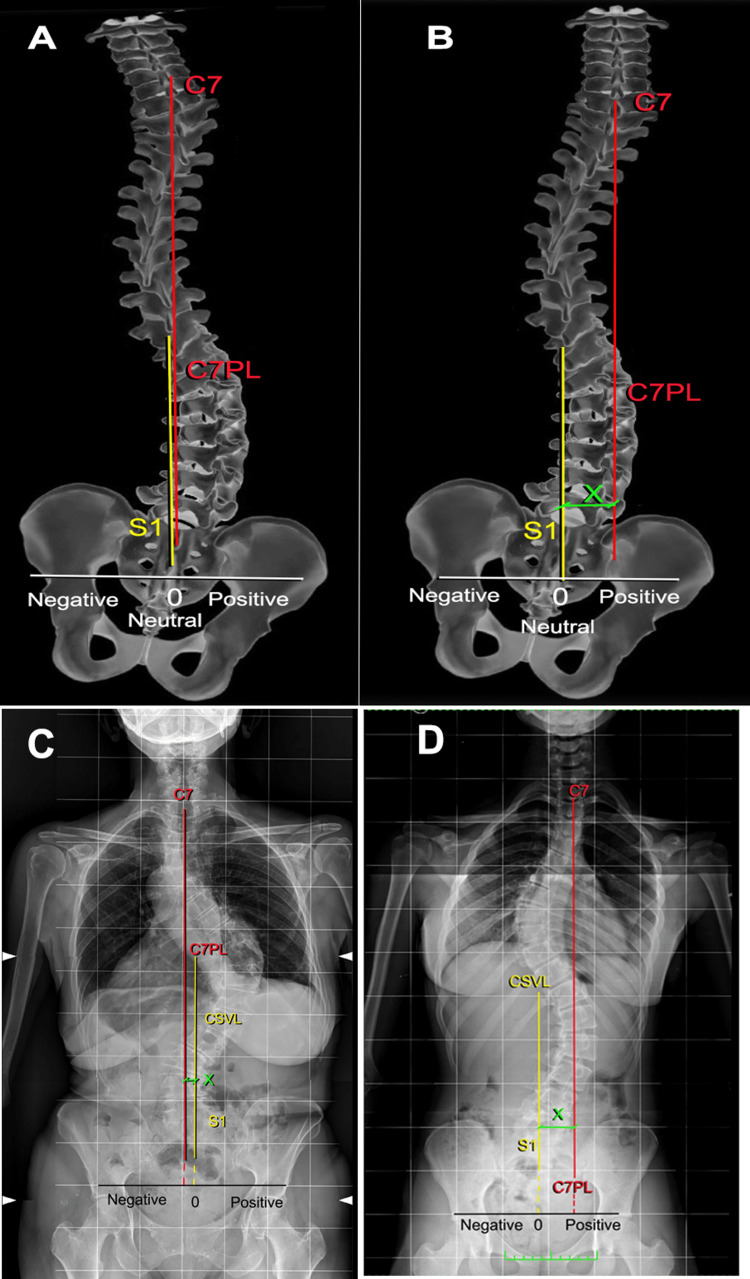
Example of the calculation of spinal balance on the coronal plane. Example of scoliotic spine with coronal compensation: A) scheme of scoliotic spine with coronal compensation; B) scheme of scoliotic spine with coronal decompensation; C) X-ray of a scoliotic patient with coronal compensation; D) X-ray of a scoliotic patient with coronal decompensation. Image Credit: Author Saverio Colonna

Before treating scoliosis with a heel lift or an entire sole lift, another important element to consider, in addition to identifying whether the discrepancy is structural or functional, is the balance of the spine on the frontal plane (coronal balance (CB)). The Scoliosis Research Society (SRS) [113] defines CB as the distance in millimeters between the vertical alignment of the midpoint of C7 with the vertical midpoint of the sacrum in the coronal plane (Figure 20); this is also reported in the literature as the Central Sacral Vertical Line often referred to by the acronym CSVL [114].

This definition adequately describes the position of the head of the body above the pelvis. According to the SRS, decompensation occurs when this alignment moves away from the midline, which is usually reported as greater than 20 mm. In the literature [114], this measurement has been called spinal balance assessment, CB, or coronal malalignment (CM). For these authors [115], it is incorrect to define CM as coronal imbalance, as referred to in the literature [114] because balance is a dynamic concept, and the full spine X-rays for spinal assessments are static rather than dynamic examinations; hence, balance and imbalance should rather be called alignment and malalignment, respectively.

Some authors [80] have used the projection of T12 on the midline of S1, while others [116] have recommended using the projection of the center of the orbits (orbital-coronal vertical axis) to insert the assessment of skull balance. For the caudal reference, however, Sangole et al. [117] recommended using the midpoint of the line that joins the center of the femoral heads, which is defined as the central hip vertical axis. These two points are comparable in healthy subjects; however, they are different in scoliotic subjects [118]. Decompensated curves, especially those in the sagittal plane, are likely to be symptomatic for low back pain [119]. The most recent work [120] relating to this topic proposed the odontoid-hip axis (OD-HA), i.e. the vertical projection of the odontoid process of the axis on the line that joins the center of the two femoral heads. In this latest work, the authors' conclusions were 1) the OD-HA analysis suggests that patients with AIS are almost three times more likely to have a misalignment compared to a population with an aligned spinal axis and 2) furthermore, coronal OD-HA analysis has a predictive value and promises to help the clinician distinguish between stable and progressive thoracic scoliosis.

A coronal imbalance greater than 30 mm was found in approximately 35% of a population of 284 scoliotic subjects aged over 50 [121]; when this imbalance was on the convex side of the curve, the results of surgical interventions were worse [121].

Notably, coronal imbalances appear to be proportionally related to the inclination of the basal vertebrae and the Cobb angle [122]. CM has received minimal attention in the literature compared to sagittal malalignment [123]. CM is evaluated in ambulatory patients in the standing position and in the sitting position for patients who cannot walk, and the condition has been well documented and analyzed in patients suffering from cerebral palsy [124]. CM, originating from spinal deformity, in this clinical situation induces an unlevel pelvis, which leads to gait disturbances in patients who can walk and to asymmetrical ischial compression as well as difficulties maintaining a proper sitting position in patients who cannot walk. CM has rarely been described, and the literature provides little guidance for surgical indications for patients suffering from adult spine deformities. However, CM is regularly evaluated in patients with scoliosis [118]. The only natural compensatory mechanism in patients with CM is contralateral knee and hip flexion, which is painful for the patient.

Additionally, unlike sagittal malalignment, which disappears in the sitting position, CM is present in the sitting and standing positions and is irrelevant only when the patient is lying down [113]. The results of CM on patient satisfaction, especially when it appears or worsens after the surgery, are not negligible. CM is rarely isolated, and it is frequently associated with sagittal plane deformities and sagittal malalignment.

**Figure 21:**
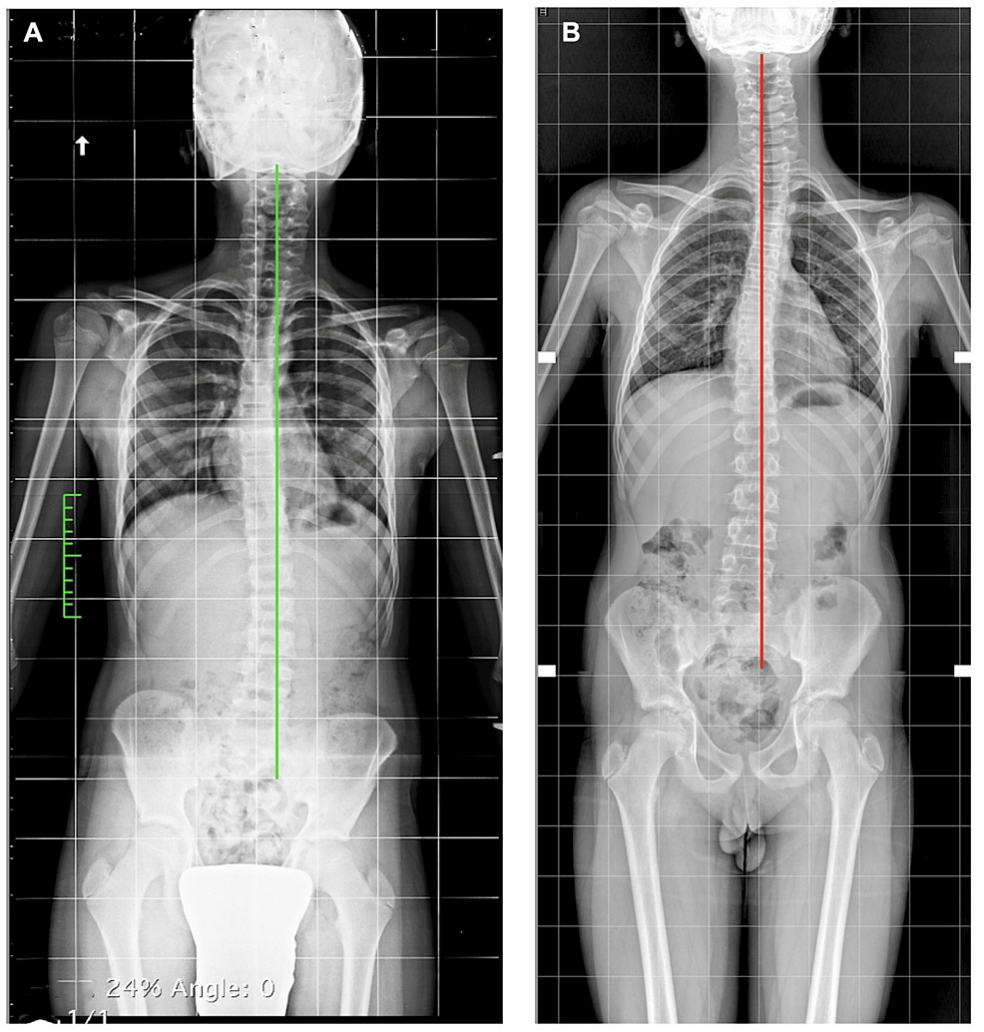
CB in different types of scoliosis. These scoliotic subjects have similar curves but opposite coronal imbalances. A) The coronal overhang and the pelvic inclination are both on the side of the shorter limb (left); B) the overhang is on the side of the longer limb (left) with contralateral pelvic inclination. Image Credit: Author Saverio Colonna

In case someone wants to treat scoliosis with a foot lift where there is an LLI with pelvic tilt on the shorter limb, one must evaluate where the projection of the T1 vertebra falls. If the CM is on the opposite side of the shorter limb (on the right in Figure 21), then compensating the LLI would worsen the amplitude of the coronal imbalance, which could cause a worsening of the scoliotic curve. The degree of imbalance on the coronal plane in the orthostatic posture could remain, although to a reduced extent, even after compensating the LLI by lifting the foot of the shorter limb [38].

**Figure 22:**
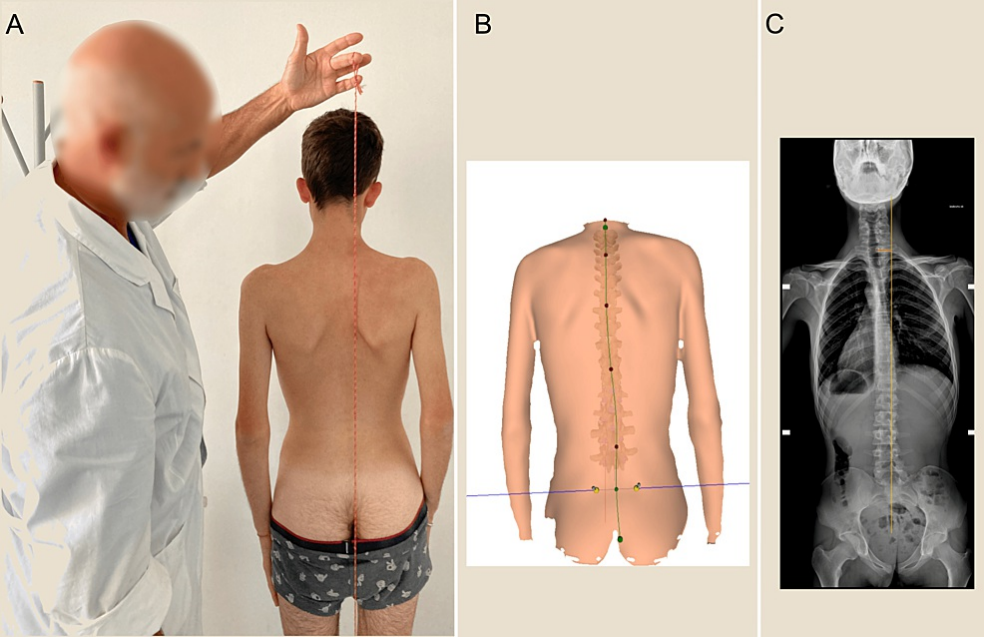
Lateral overhang evaluation methods. The lateral overhang can be evaluated by using a plumb line (A), spinometric examination (B), or orthoroentgenogram (C). Image Credit: Author Saverio Colonna

To assess CM, the most suitable test is the orthostatic orthoroentgenogram of the entire pelvis and spine, but useful indications can also be obtained via rasterstereography or a plumb line assessment (Figure 22). The advantage of radiography is precision; the disadvantage is the use of ionizing radiation.

Using rasterstereography has the disadvantage of increasing the cost of the examination, although it may be a worthwhile compromise because of its degree of precision [125] without harmful effects. This allows multiple evaluations to be performed to determine, for example, the best structure by using a series of increases under the foot. In the literature [94,126], a correlation among the LLIs, pelvic obliquity, and the inclination of the trunk in the coronal plane (overhang) is reported.

**Figure 23:**
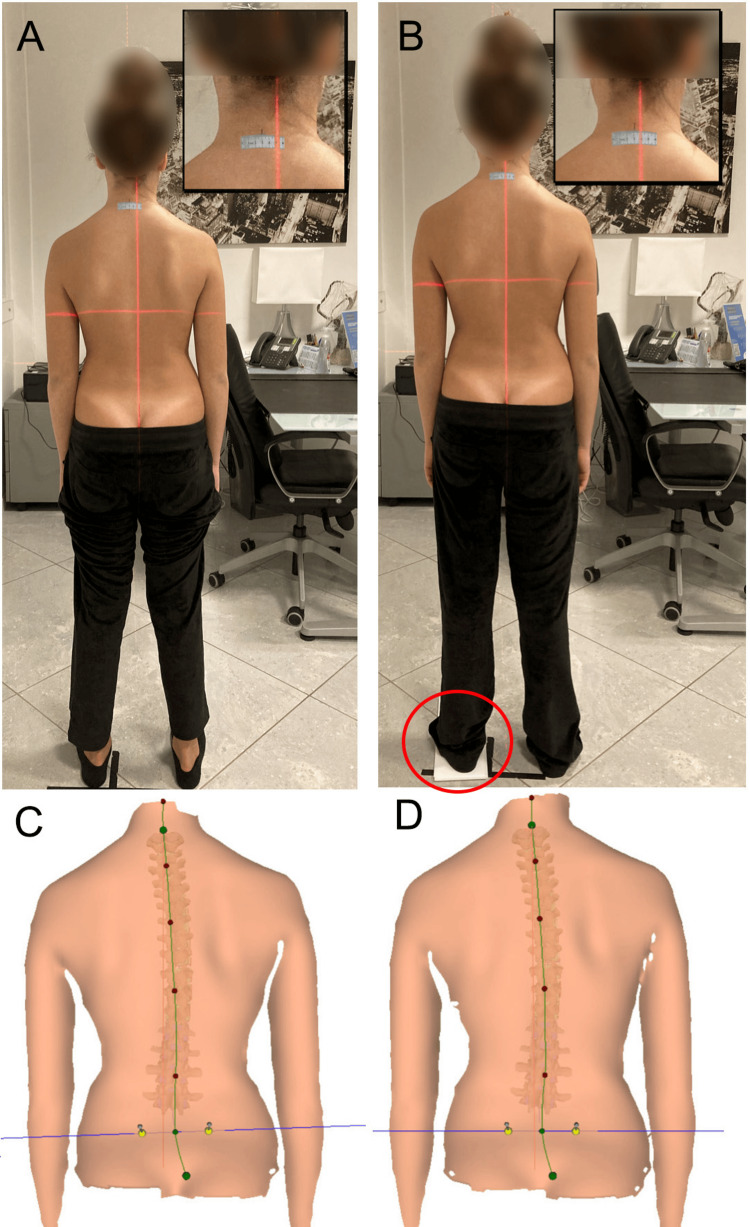
Evaluation of the coronal plane balance of a scoliotic subject. This series of images presents an example of a CB evaluation. In the top row, a laser beam is used (A, B) (in the inset box, the images of the C7 spine are marked with a pen and ruled paper as well as a laser beam). The bottom row illustrates a spinometric evaluation (formetric) (C, D). The left images (A, C) are in neutral condition, whereas the right images (B, D) include a 10 mm elevation under the left foot. Image Credit: Author Saverio Colonna

The plumb line and the laser beam are both inexpensive and widely available, and using a simple ruler or a small piece of ruled paper glued to the skin with bioadhesive, as illustrated in Figure 23, allows a quantitative evaluation; the disadvantage, however, is imprecision.

Figure 23 presents an example of a laser beam evaluation of the coronal overhang on a patient with a left dorsal-lumbar scoliotic curve (D10-L4) of approximately 16° Cobb and a right dorsal curve (D3-D10) of 15° Cobb. Figure 23 also offers a spinometric evaluation of the same patient. The procedure begins by highlighting C7 and S1 via a vertical mark as the patient stands barefoot (image on the left). A small piece of ruled paper is applied with bioadhesive (in the image, the alignment of the paper with respect to the spinous process of C7); the laser beam is in self-leveling mode and is aligned with the vertical axis on S1 (intergluteal fold) while the millimeters of the deviation from the spinous process of C7 and the laser light, indirect reference of S1, are noted. In the second evaluation, a riser is inserted under the foot (image on the right) while the patient moves as little as possible, and the extent to which the laser beam moves with respect to the paper is reevaluated. The rise reduces the alignment of the laser from the initial 5 cm to 4 cm; therefore, the overhang is reduced by 10 mm. In the bottom row of images, the same subject is evaluated with spinometry (formetric), and the rise reduced the obliquity of the axis passing through the PSIS and the left coronal overhang (vertical red line).

Treatment

In clinical practice, scoliotic patients with an asymmetry of the pelvis are common. Customized foot orthoses may be considered to manage mild idiopathic scoliosis in young patients; evidence, however, is poor, and quality studies are needed to validate these findings. It is difficult to decide whether leveling should be achieved by lifting the sole or placing orthoses inside the shoe because the literature reports that the asymmetry may be compensatory rather than causal [30,31].

Some researchers, in fact, have indicated that correcting the LLI levels pelvic imbalances and reduces the angle width of functional scoliosis [23,38,53,106], whereas others have demonstrated that artificially generated LLIs have a small, acute effect on the curve deformity of the lumbar spine [103,127-129]; however, these studies used rasterstereography to evaluate the deformations that were induced by artificially lifting the foot, which has acceptable reliability only for curves on the sagittal plane (lordosis and kyphosis) but is less reliable on the coronal and transverse planes. One of these studies [128] concludes that the induced dysmetria modifies the balance of the pelvis (inclination or torsion) but not the lumbar curve; however, the authors refer to the sagittal curves (lordosis and kyphosis), which are known to be affected by flattening only in an advanced stage [130].

Another work [127] in which rasterstereography was used as an investigation tool presents changes in the torsion or inclination of the pelvis and rotation or inclination of the lumbar vertebrae upon artificial lifting of one of the two limbs by 10 mm, 20 mm, and 30 mm; the induced adaptations do not appear to differ with age.

Scoliosis that develops due to the discrepancy in the length of the lower limb is included in functional scoliosis; in fact, this type of scoliosis reverses totally or partially when its cause, the LLI, is removed [35]. It is intuitive that regression is only possible if the spinal alterations have not become structured [75,88].

Klein et al. [131] support the persistence of pelvic torsion in subjects with an anatomical LLI, reporting that the alteration persists in the upright and sitting positions. When pelvic torsion remains without the subject's weight being loaded on the femoral heads, this indicates that the torsion has been incorporated into the joints as a normal position. Rhodes et al. [132] find that the side and width of the shorter leg in the prone and especially supine positions are not significantly correlated with the radiographic anatomical LLI, thus indicating that these are separate phenomena. The pelvic joints, ligaments, and muscles adapt to the anatomical LLI, consequently making any twist structural. This presumed biomechanical adaptation makes unloading limb alignment asymmetry tests unreliable as a measure of an anatomical LLI [133].

Equalizing limb length is a difficult task that requires careful thought, planning, and an appropriate management strategy [134]. In many cases, the causes of LLI development, the prognostic progression of the discrepancy, and the patient's general and mental condition are essential factors in the selection of therapeutic options [135]. The most important factors that should be considered in planning are the precisely measured length discrepancy, an in-depth analysis of the relationships between the differences in levels of the limbs, pelvis, sacrum, and last lumbar vertebrae, and the patient's age.

**Figure 24:**
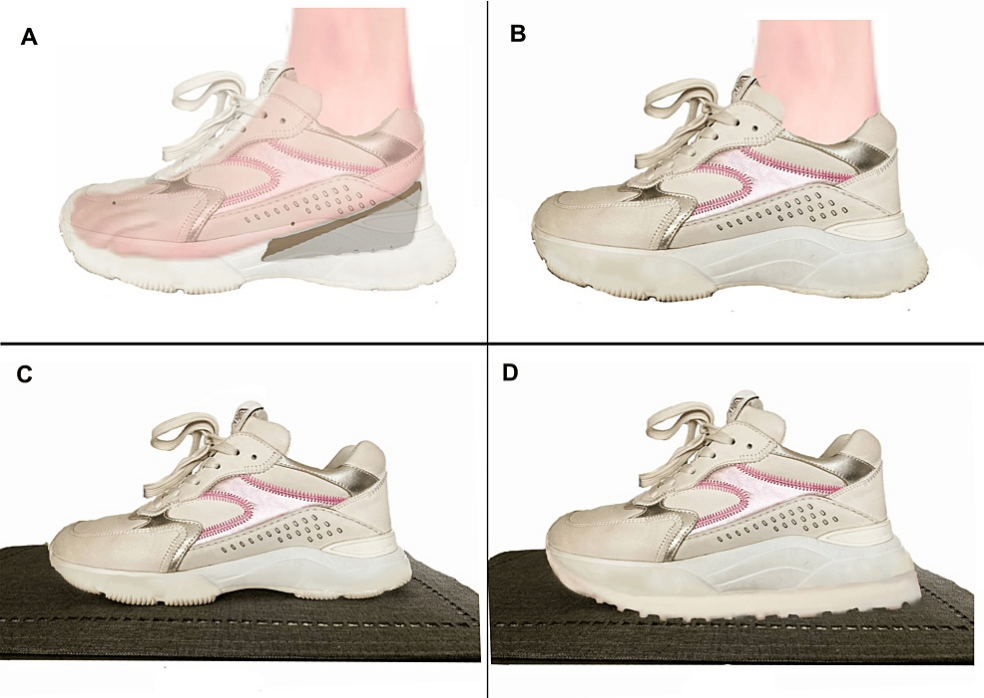
Examples of lifting the heel Examples of lifting the heel by raising it inside the shoe (A, B) and outside of the shoe (C, D).

LLIs of ≤ 2 cm are believed to rarely cause structural problems, and internal shoe lifts may be added with a thickness corresponding to the LLI [3,15,34]. The most commonly used internal lifts are 0.5 cm to 1.5 cm thick (Figure 24A, 24B). For LLIs of 1.5 cm to 2 cm, it is often more comfortable for the patient to use an external sole or heel to lengthen the limb (Figure 24C, 24D).

For differences from 3 cm to 20 cm, surgical compensation is recommended. LLIs of 3 cm to 5 cm in subjects still in the growth phase are usually treated by attempting to shorten the longest limb through growth arrest (epiphysiodesis). LLIs of 6 cm to 15 cm require surgery to lengthen the shorter limb and the postsurgical use of external fixators. Treatment of an LLI > 15 cm requires a combination of shortening the longer limb and lengthening the shorter one. Surgical treatment of an LLI > 20 cm does not offer many possibilities for complete leveling of limb length. In these conditions, external prostheses are used. If full compensation is not achieved after surgery, then the difference should be compensated with an internal or external heel lift of the shoe [6, 113]. The improvement in LLIs, the reduction in the difference in limb lengths, is notable between pre- and post-surgery in patients who have undergone scoliosis surgery; this improvement, however, is limited to patients who had a shorter left limb [82]. The authors posited that this result suggested the existence of different mechanisms between the shortening of the right and left limbs.

Patient age is the second most important element to consider when selecting treatment options. For children and adolescents who have not stopped growing, there is a change that could be yet to happen in the use of compensatory and developmental mechanisms of the body. The decision to use conservative (internal or external shoe lift) or surgical (epiphysiodesis) methods is based on the possibility of stimulating or inhibiting limb growth. Conservative treatment can be applied to subjects who have completed growth by externally equalizing the asymmetry. Lasting correction, although perhaps not complete correction, can be achieved in such cases only with surgical treatment by shortening or lengthening the limbs [91,115].

## Conclusions

Scoliosis represents a pathology whose cause is still not known in most cases. Very often the alteration of the spine is associated with a structural difference in the length of the lower limbs. If it would be natural to think of compensating, with increases under the foot of the short limb, all the cases in which this association is encountered; before doing so, it is advisable to carry out a balance assessment in the coronal plane. For this balance, the distance from the vertical projection of C7 should be taken into consideration; probably it could also be useful from the cranial base or inter orbital point, when present in the radiograms, compared to the sacral base or the projection of the line joining the center of the femoral heads.

To evaluate the alignment in the coronal plane, radiograms can be used, which represent the most precise method, but other solutions, such as spinometry and laser beam, can also be used with a good approximation.
