# Correction: Scoliosis and Lower Limb Inequality: To Lift or Not to Lift, That Is the Question 

**DOI:** 10.7759/cureus.c170

**Published:** 2024-04-29

**Authors:** Saverio Colonna, Fabio Casacci, Corrado Borghi

**Affiliations:** 1 Rehabilitation Medicine, Spine Center, Bologna, ITA; 2 Research and Development, Osteopathic Spine Center Education, Bologna, ITA

This article has been corrected by the Editor-in-Chief to fix an incorrect citation. In reference 25, the authors mistakenly cited the correction notice instead of the article itself. As this article references information and results from the article, not the correction, we have updated reference 25 to accurately cite the article in question. The authors and journal regret that this error was not identified and resolved prior to publication.

